# Using Classical Population Genetics Tools with Heterochroneous Data: Time Matters!

**DOI:** 10.1371/journal.pone.0005541

**Published:** 2009-05-14

**Authors:** Frantz Depaulis, Ludovic Orlando, Catherine Hänni

**Affiliations:** 1 Laboratoire d'Ecologie et Evolution, CNRS UMR 7625, UPMC Paris Universitas, Ecole Normale Supérieure, Paris, France; 2 Institut de Génomique Fonctionnelle de Lyon, Université de Lyon, Université Lyon 1, CNRS, INRA, Ecole Normale Supérieure de Lyon, Lyon, France; University of Montreal, Canada

## Abstract

**Background:**

New polymorphism datasets from heterochroneous data have arisen thanks to recent advances in experimental and microbial molecular evolution, and the sequencing of ancient DNA (aDNA). However, classical tools for population genetics analyses do not take into account heterochrony between subsets, despite potential bias on neutrality and population structure tests. Here, we characterize the extent of such possible biases using serial coalescent simulations.

**Methodology/Principal Findings:**

We first use a coalescent framework to generate datasets assuming no or different levels of heterochrony and contrast most classical population genetic statistics. We show that even weak levels of heterochrony (∼10% of the average depth of a standard population tree) affect the distribution of polymorphism substantially, leading to overestimate the level of polymorphism *θ*, to star like trees, with an excess of rare mutations and a deficit of linkage disequilibrium, which are the hallmark of e.g. population expansion (possibly after a drastic bottleneck). Substantial departures of the tests are detected in the opposite direction for more heterochroneous and equilibrated datasets, with balanced trees mimicking in particular population contraction, balancing selection, and population differentiation. We therefore introduce simple corrections to classical estimators of polymorphism and of the genetic distance between populations, in order to remove heterochrony-driven bias. Finally, we show that these effects do occur on real aDNA datasets, taking advantage of the currently available sequence data for Cave Bears (*Ursus spelaeus*), for which large mtDNA haplotypes have been reported over a substantial time period (22–130 thousand years ago (KYA)).

**Conclusions/Significance:**

Considering serial sampling changed the conclusion of several tests, indicating that neglecting heterochrony could provide significant support for false past history of populations and inappropriate conservation decisions. We therefore argue for systematically considering heterochroneous models when analyzing heterochroneous samples covering a large time scale.

## Introduction

**Figure 1:**
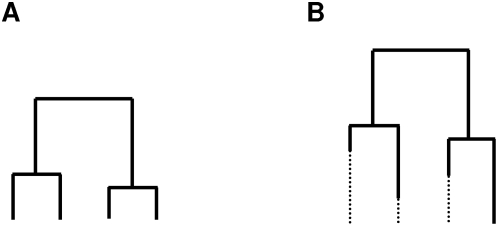
Heterochrony effects on gene genealogies. (A) Contemporaneous dataset. (B) Heterochroneous dataset. Lineages of sequences cannot reach a common ancestor before they are contemporaneous.

Most present population genetics analyses rely on coalescent theory, representing the genetic history of a random set of gene copies with genealogical trees where nodes represent coalescent events, that is when two evolutionary lines of descent reach a common ancestor [1] (Figure 1A; see Table 1 for a summary of notations). This sampling theory allows an efficient treatment of data and overall good predictions for the outcome of evolution on a set of gene copies in population(s) under specific demographic and migration scenarios. It is most simply used within the framework of the classical population genetics Wright-Fisher model (WF) [2] (hereafter “standard”) and one of several implicit assumptions is that all individuals are sampled at the same time (hereafter “contemporaneous”). This is reasonable for most datasets sampled on extant species since (1) polymorphism arises from mutations occurring along the total size of genealogies (i.e., the sum of all branch lengths) and (2) the number of generations covered across the sample is low with regard to the total depth of the genealogy (the root of the tree, MRCA for “most recent common ancestor”) that lasts on average 2 *N_e_* generations for mitochondrial DNA with *N_e_*, the effective size of (females for) the population considered, assumed to be large in the coalescent framework.

**Table 1:** Summary of the main statistics, parameters and notations.

Statistics/parameter	Definitions	Ref.
WF/*standard*	Wright Fisher population genetics model assuming in particular a constant size, well mixed neutral population.	
*N_e_*	Effective population size: equivalent size for an ideal WF population. The relevant time scale for population genetics processes is in *N_e_* generations units.	
MRCA	Most recent common ancestor, root of an intraspecific tree.	
IMSM	Infinitely many site mutational model adapted to nucleotide polymorphism, sequence data.	[41]
*θ*	Mutational parameter of the population θ = 2*N_e_*μ (with an additional factor two for diploid data).	
*n*	Sample size, subscript ‘*i*’ refers to a time subset, ‘*A*’ and ‘*B*’ to (geographical) subpopulations.	
*t_i_*	Time to the subset *i*.	
*S*	Number of polymorphic sites.	[41]
*θ_W_*	Watterson's estimator of *θ*, proportional to *S* and corrected for sample size.	[41]
*π*	Diversity estimator of *θ*: average difference between pairs of sequences; subscript ‘*b*’ refers to between populations, ‘*h*’ to heterochrony corrections, ‘*μ*’ based on mutation rate estimate.	[42]
*D_a_*	Nei's net distance between two populations *π_b_*–*π* with *π* the average within population diversity.	
*F_st_*	Population differentiation (genetic distance) index *F_st_* = *D_a_*/*π_b_*.	[43], [44]
*star* scenario	scenario leading to star shape of genealogical trees, with long external branches: strong bottleneck, population expansions, recent fixation of a closely linked advantageous mutation (selective sweep) or complex population structure such as a collection small samples from a large number of populations.	
*balanced* scenario	leading to balanced tree with long internal branches: population contraction, simple population structure between a small number of population, each with similar, substantial sampling effort.	
*D_T_*	Tajima's *D* sensitive to the proportion of rare mutations, negative for star scenario.	[39]
D^*^	Fu and Li's D^*^ related to the proportion of unique mutations (singletons) (oriented), negative for star scenario.	[40]
*H_FW_*	Fay and Wu's *H* positive selection test for partially linked advantageous mutation, sensitive to the proportion of frequent mutations (derived, oriented), negative for corresponding asymmetric trees.	[49]
*K*	Number of haplotypes elevated (with respect to *S*) for star scenario.	[51]
*H*	Haplotype diversity (sensitive to their frequency) elevated for star scenario.	[51]
*LD*	Linkage disequilibrium: statistical association of mutations, trends of various mutations to be carried by the same individuals.	
*Z_nS_*	Average LD between pairs of polymorphic sites measured through allelic correlation *ρ*, reduced for star scenario.	[50]
Pearson *r*, slope	Recombination test: correlation between pairwise LD (*ρ*) and the distance between mutated sites	[53]

Sampling across a few generations may be very useful in particular to estimate the effective population size from the fluctuations in allele frequencies. On such limited time scales, mutations may be neglected (see e.g. [3], [4]). With more heterochroneous datasets however, mutations occurring between sampling points cannot be neglected compared to those occurring on the whole tree. Such a situation occurs in “measurably evolving populations” [5] of microorganisms (e.g., viral datasets), where generation times are short and samples are collected at different stages of the disease [6] or from different historical epidemics [7]. It is relevant for experimental molecular evolution on bacteria as well, since post-treatment subsets are generally compared to the initial state [8]. Clearly, ancient DNA (aDNA) datasets, which exhibit haplotypic information over thousands [9]–[13] to tens of thousands of years [14]–[19], intrinsically violate the constant sampling time assumption. An illustrative case we use below is that of one extinct species, namely the Cave Bear (*Ursus spelaeus*), a species that inhabited Europe from 300 to 12 thousands of years ago (KYA) [20], [21] and for which a large set of heterochroneous mtDNA control region (CR) sequences have already been reported [22]–[28].

Compared to entirely modern datasets, heterochroneous ones can provide additional valuable information about the history of the species by adding known states along the genealogy of a sample. However, such data require taking heterochrony explicitly into account in the analyses (see Text S1 for an overview of related methods and a justification of the present strategy). To date, heterochroneous simulation-based methods have been used to analyze heterochroneous data and provide estimates of mutational parameters, MRCA and radiation dates [29], [30]; generation time [31]; effective population size, complex population structure and demographic history [9], [32], [33] and its correlation with climate change (the “phylochronology” approach described in [10], [34], [35]; the “skyline plot” used in [36]). Heterochroneous simulations were also used to test for local contribution of past populations to current polymorphisms (e.g., isolation *vs* immigration hypotheses; [37]). Finally, Achaz *et al* used a population differentiation test to detect within-host temporal evolution of HIV populations [38].

But so far, despite such interesting practical applications, to what extent classical population statistics and tests are sensitive to heterochrony has not been specifically addressed. Though largely ignored in the literature, this issue may well bias population genetics analyses, if neglected. Since in the coalescent process, mutations are uniformly mapped conditionally on an independently drawn genealogy [1], the differences in branch lengths resulting from heterochrony should affect the polymorphism pattern of simulated datasets (Fig. 1). Therefore, whether (and if so, to what extent) heterochrony in the data affects the population genetics analyses needs to be addressed.

To estimate the bias induced by heterochrony in standard analyses, we focus on summary statistics that describe some key features of the data at the expense of some loss of information. They include the so-called neutrality tests, which are generally used as an index of selection or demographic patterns and can be considered as a first step toward a deeper understanding of the history of a population. Heterochrony effect on the tree shapes should in turn affect for instance the frequency distribution of mutations, on which many neutrality tests rely (e.g., Tajima's *D* [39], and Fu and Li's statistics [40]).

Here, we contrast simulation outcomes from classical (contemporaneous) and heterochroneous coalescent models to address how much estimators of polymorphism levels, divergence and neutrality tests are affected by heterochrony. We show that if not corrected, heterochrony introduces substantial biases in population statistics. Defining simple corrections, we further illustrate how these effects could affect the inference of qualitative history of past populations, using the extinct Cave Bear from the Pleistocene period as an example.

## Methods

### Simulations algorithm

We used standard coalescent techniques following Hudson [1]. The proportion of significant simulations was sorted depending on whether there was a deficit or an excess of the statistics. Times are expressed in units of 2*N_e_* generations (i.e. average *t_MRCA_* for mitochondrial sequences which include most aDNA datasets), the relevant evolutionary time scale for intraspecific molecular evolution.

From now on, “subset” refers to a set of sequences from a particular time (or time range when faced to uncertainty) and “subsample” to a time-independent partition of the data (e.g., according to geographic repartition). The main modification introduced here is to allow for heterochrony between sequences, as defined by a subset of (*t_i_, n_i_*) couples, with *t_i_* the time to a contemporaneous subset and *n_i_* the number of sequences involved (Fig. 1, Fig. S1). The process begins with the most recent subset of sequences (*t*_0_ = 0, *n*_0_) and allows coalescence between them (this is classically modeled by exponential probability law with parameter *n*_0_(*n*_0_–1) [1]). Older sequences are included step by step when reaching their sampling times (see figure S1 and text S1 for details). The process is repeated until all sequences are incorporated and the root of the tree is reached.

### Statistics considered

Traditional population genetics statistics first include index of the level of genetic variation, estimators of the mutational parameter of the population (polymorphism *θ* = 2*N_e_* μ, for haploid loci), reflecting the compound effect of the mutation rate and the effective size. We consider the two simplest and most popular estimators, *θ_W_* based on the number of segregating sites in a sample [41], proportional to the total size of the tree, and *π*, the average number of differences between two random sequences of a sample [42]. Other statistics include tests of population differentiation (*F_st_*, reflecting the level of genetic isolation between populations). We use the *F_st_* statistic of Hudson, Slatkin and Maddison [43] adapted to sequence data and tested according to a procedure involving permutation (randomization) of individuals between populations (with 500 permutations) [44]. We addressed whether different temporal sampling schemes between two subsets could lead to some apparent population structure. We used simulations of a single panmictic (well mixed) population from which two subsets were drawn, typically with different serial sampling schemes. Finally, we considered several commonly used neutrality tests previously reviewed in [45]. In fact, they test for a full (WF) neutral model with all its assumptions including neutrality, but also strong assumptions about demography and the mutational model. Here we use the infinitely many site model (IMSM [41]), generally used for nucleotide polymorphism data and assuming in particular the absence of recombination and that each new mutation affects a previously non-mutated nucleotide site, thus implying the absence of multiple hits and homoplasy (recurrent mutations occurring on the same nucleotide site, the rational being that most site usually show no variation- but see the discussion). In the present paper, we address if heterochrony within a dataset, if not explicitly taken into account, can also be considered as a relevant alternative when rejecting the null model.

Tests were chosen so as to limit redundancy and are based a priori on various sources of information. The first category (1) sums up various aspects of the frequency distribution of mutations in the dataset, i.e. the *frequency spectrum of mutations*, how many mutations hit one, two, three, *i*… individuals; the various polymorphic sites being considered independently. These include the *D* statistic of Tajima [39], assuming non-oriented mutations, i.e. that for each variable site along an alignment of sequence, we do not know which one is the ancestral state and which one is the newly arisen mutant, we thus consider only the frequency of the rarest of the two variants. A negative *D* indicates an excess of low frequency variants, reflecting a star like genealogy which can result from for instance population expansion possibly following a drastic bottleneck [39], [46] or recovery of variation after the spread of a tightly linked advantageous mutation [45], or a mix of a number of populations each with low sample sizes [47] and other complex compounds of population structure with demography: such as extinction recolonization processes [48] (hereafter *star* scenarios). Distinction between those scenarios should be made on other grounds. On the contrary, a positive *D* reflects a balanced tree with long internal branches. This pattern can result from population contraction, moderate bottleneck (of small magnitude and short duration) or a mix of two genetically isolated stable populations with similar sampling effort (this mix could be artificial from sampling collection - *stratification* - or reflect real natural population admixture; hereafter *balanced* scenarios). The D* of Fu and Li [40] reflects the proportion of singletons (mutations carried by a single individual in the sample) compared to intermediate frequency ones. A negative D* reflects an excess of singletons reflecting star scenarios as for the previous statistic. Other frequency distribution statistics (including other statistics from [40]) use oriented mutations: they consider the frequency of the derived state, with ancestral states deduced from an outgroup (a closely related species). The *H* statistic of Fay and Wu [49] (hereafter *H_FW_*) reflects the proportion of high frequency derived mutations. It can also be interpreted in terms of how symmetrical the tree is, whether the numbers of descendants on each side of the most internal nodes are well equilibrated. A negative *H_FW_* indicates an excess of high frequency derived variants and an asymmetrical (unbalanced) tree which is typical of the spread of selectively favored mutations on a partially linked locus [49].

The second category (2), though not independent of the frequency of variants, also reflects if several mutations tend to affect the same individuals (i.e. the linkage disequilibrium structure) either through comparisons of pairs of variable sites (mean allelic correlation *Z_nS_* [50]) or global association on the entire alignment *via* haplotype statistics: haplotype number *K* and haplotype diversity *H*, the latter taking into account the frequencies of haplotypes [51]. Low values of those two statistics with respect to the number of polymorphic sites and the number of sequences in the dataset indicate a strong global linkage disequilibrium structure (hereafter “haplotype structure”). In the absence of recombination, linkage disequilibrium measures tend to reflect the shape of the tree (the tendency of mutations to appear on related branches [52]) and maximal absolute values of linkage disequilibrium tend to decrease with decreasing frequencies of the mutations. Thus, relatively high linkage disequilibrium values (low *K*, *H*) reflect balanced trees (and scenarios) whereas low values reflect star scenarios (see [46], [51] for details and a graphical description). Finally, we included a measure of the correlation between linkage disequilibrium and the distance between mutations along the sequence. This test was used to detect putative recombination in mitochondria [53] (see discussion about this peculiar side issue). We checked that it was not affected by heterochrony. We use Pearson's correlation coefficient between pairwise linkage disequilibrium (as measured by allelic correlation), tested by permutations between sites, following the original procedure (singletons, which show little information about recombination and add noise, were removed from the analyses).

### Cave Bear application dataset

All Cave Bear DNA sequences included in our dataset have been reported elsewhere, with different combinations of PCR primers [22]–[28] (Table S1, Fig. S3). The total dataset constitutes a 322 bp alignment of 118 sequences with 45 polymorphic sites. As a result, the different sequences do not exhibit similar lengths, resulting in a substantial amount of missing data (see Figure S3 for an alignment of polymorphic sites). We did not restrict the analysis only to the longest region covered for all haplotypes but generated various partitions of the data in order to use most of the information available. In particular, the “Scladina” subsample consisted of a reasonably large (20 sequences) local sample, derived from a single deposit in Belgium (Scladina cave), covering a wide time range (30 –130 KYA) [54], with all of the sequence times being reasonably accurately estimated by precise stratigraphic records (for details, see [54] and the Appendix section provided in [28]). The other 98 sequences were sampled throughout Europe, north and south of the Alps (Austria, Croatia, France, Germany, Italy, Slovakia, Slovenia and Spain). The ages of the different sequences were estimated according to radiocarbon dates or precise stratigraphic information (49 and 25 sequences, respectively).

For the heterochroneous analyses, estimates of *N_e_* and generation times are required to scale times in units of 2*N_e_* generations. We used a rough estimate of *N_e_* derived from [25] which should be sufficient to estimate the magnitude of the biases (see text S1 for details).

Uncertainty in the estimation of *N_e_* can be translated into uncertainty in time estimates. For the time uncertainty analyses, we allowed the age of *N_e_* to vary uniformly within the limits of the range estimated from [25]. We applied an equivalent procedure for the generation time on their prior range (10–17 years).

## Results

### Simulations

**Figure 2:**
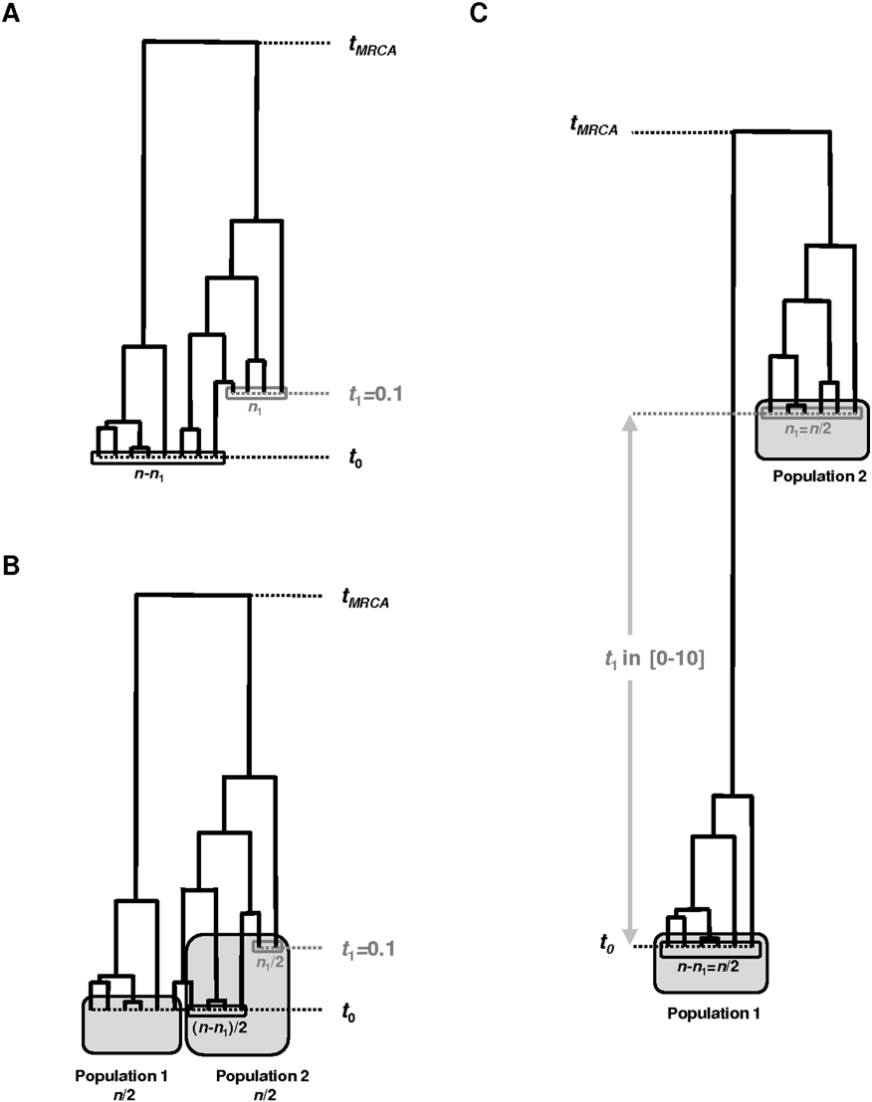
Outline of the main models simulated. (A) Single panmitic population with variable proportions of ancient data (*n*_1_/*n*) (one third in this example) of moderate age (0.1) with respect to the time unit of 2*N_e_* generations (i.e. the average age of the root of a population tree in the homogeneous, contemporaneous case; see figure 3). (B) Corresponding simulations for the population differentiation (*F_st_*) analysis; a single homogenous set of individual, but labeled as randomly split into two populations equally sampled, one showing variable proportions of ancient data (*n*_1_/*n* again, one third in this example) of moderate age (again *t*_1_ = 0.1; see figure 3, *F_st_*). (C) Single panmitic population with equal proportions of ancient and modern data (*n*_1_/*n* = 1/2, equivalent to the two population samples for the *F_st_* analysis;), the age of the ancient samples ranges from 0 to 20 *N_e_* generations; see figure 4). See text for more explanations.

The simulation approach is used to address the properties of the sample summary statistics when faced with heterochroneous data. When statistical tests are used classically, assuming no heterochrony in the data, are these tests still robust? Do we see apparent evidence of selection or demographic variation as an artifact of serial sampling? Here, we aim at describing the general pattern and at giving clues on how to interpret the statistics, rather than being more exhaustive about the set of parameter values and sampling scheme. For simplicity, we restrict the presentation of most of our results to two sampling times *t*_0_ = 0 and variable *t*_1_ (counted backward in time), and the corresponding two subset sizes (*n*–*n*_1_ and *n*_1_, respectively; see figure 2 for the design of the simulated conditions). For illustration purposes, we focus on parameter values (*n* = 100, *θ* = 4) on the order of magnitude of those of the application case below. A much broader set of parameter values was investigated and showed qualitatively consistent results (results not shown).

### Polymorphism and genetic diversity

**Figure 3:**
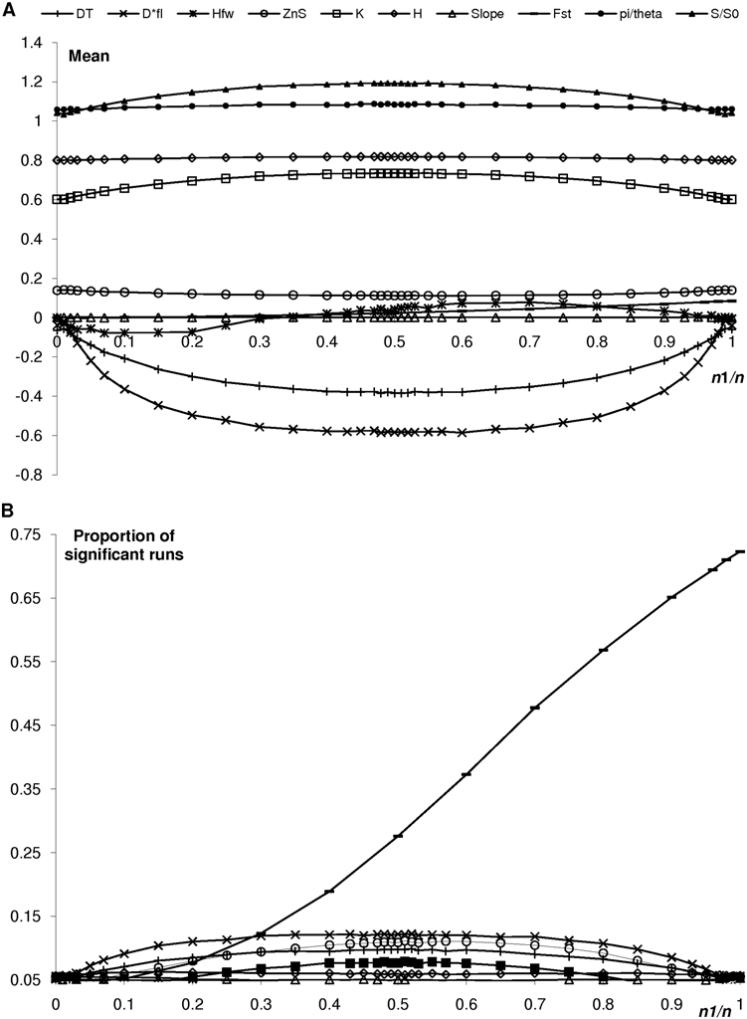
Effect of subset size on statistical tests. The temporal spacing between the two subsets is set to 0.2 *N_e_* generations. Ten thousands runs were simulated for each set of parameter values. The *X* axis corresponds to the proportion of the ancient subset. DT: Tajima's *D* [39]; D*FL: Fu and Li's D* [40]; HFW: Fay and Wu's *H* [49]; Note that this statistics is not standardized by its variance and can thus potentially show high absolute values, hence a rather erratic behavior on fig. 2a]; ZnS: Kelly's *Z_nS_* [50]; K and H: Depaulis and Veuille's haplotype tests ([51]; *K* is scaled to the expected *S*+1, its expected maximal value in the absence of recombination and homoplasy); Slope: recombination test, pearson correlation coefficient between pairwise allelic correlation and distance between mutations tested by permutations according to Awaddala and colleagues [53]; Fst: Hudson, Slatkin and Maddison's *F_st_* [31] between two population subsamples of equal size 50∶50, then the *X* axis corresponds to the proportion of ancient sequences in the second subset. This *F_st_* is tested by permutations between subsets [33]. Five hundred permutations were used in these last two tests. (A) Mean (bias) and (B) Proportion of significant runs that show deviation from the standard coalescent expectations (rate of false positives). Only portions of curves above 6% (as an arbitrary threshold of marginal significance) are shown for clarity. Note the different scale of the *Y* axis on the top part of figure B. Empty symbols: deficit of the statistics; filled symbols: excess of the statistics.

**Figure 4:**
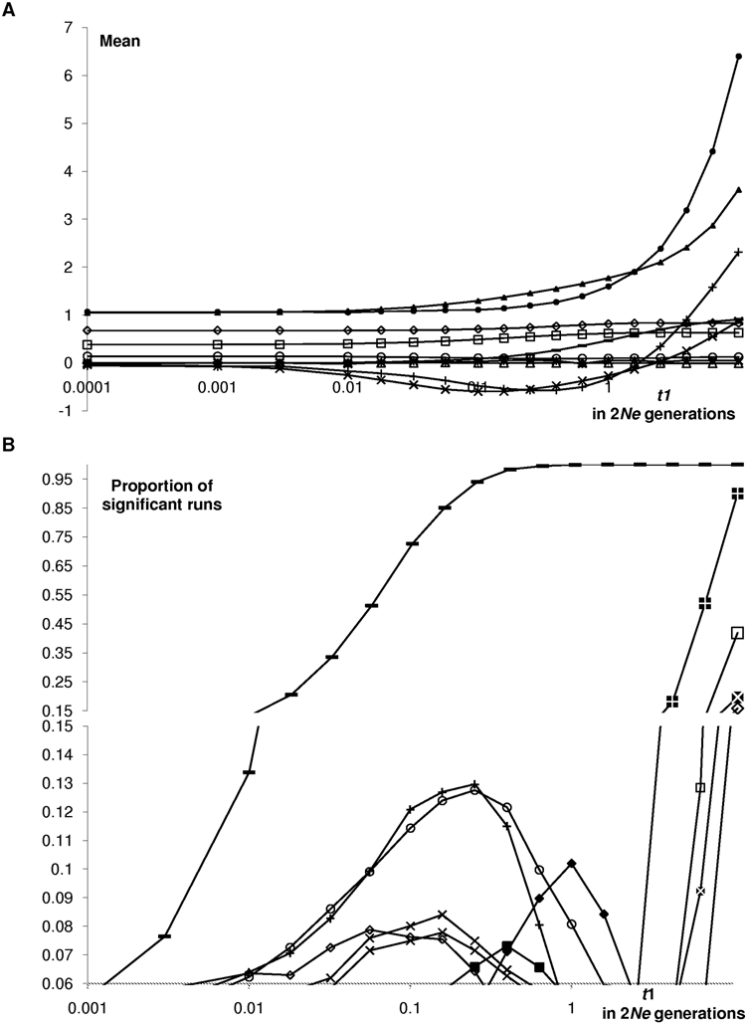
Effect of the time spacing with a 50% subset on statistical tests. *n_i_* = 50, whole second population subsample in the *F_st_* analysis. The *X* axis is expressed in units of 2*N_e_* generations. Same labeling and other parameter values as in figure 3.

Figure 3 shows the effect of the proportion of ancient data in the sample (*n*_1_/*n*) on the statistics, for moderate time lapse between the two subsets (*t*_1_ = 0.2 *N_e_* generations, i.e., 10% of the average MRCA of a contemporaneous homogeneous population; Fig. 2A). Figure 4 shows the effect of the time lapse *t*_1_ on the statistics with half of the data being ancient (*n*_1_ = *n*/2, Fig. 2C). Results for unbalanced sampling proportions – *n*_1_/*n* = 10% and 90% – are shown in Fig. S4). The top parts (A) of those figures show the effect on the means (average bias) and the bottom parts (B) on the proportion of significant runs (of false positive tests) when compared to the standard, contemporaneous, case, both tails being considered separately (empty and filled symbols are used for a deficit and an excess of the statistics, respectively). The effect of the temporal spacing on the expectations of the statistics is noticeable for a broad range of parameter values (Fig. 3–4). The most striking effect is that heterochrony systematically tends to overestimate the level of genetic polymorphism (Fig. 3A and 4A; note that under a classical coalescent model, *π* and *θ_W_* should provide unbiased estimates of *θ* for contemporaneous data, standardized *π* and *θ_w_* are therefore expected to equal 1 on average – leftmost and rightmost points on Fig. 3A, leftmost one on Fig. 4A – and any deviation from this value can be attributed to heterochrony).

The deviation increases dramatically with time spacing between sampling points (Fig. 4). For *t*_1_ values inferior to 2*N_e_*, the effect is greater for *θ_W_* (19% increase for *t*_1_ = 0.1, *n*_1_ = *n*/2; Fig. 3A, see ‘S/S_0_’), which is more sensitive to the shape of the tree than π (8% increase; see ‘pi/theta’). This effect increases dramatically with the time lapse (similar to simple population structure effect, Figure 1c and 3a). For 2*N_e_* generations, 59% bias for π and 77% for *θ_W_* (Fig. 4A); for 10×2*N_e_* generations and two subsets of equal sizes, *π* is increased by a factor of 6 (Fig. 4A). In such cases, that led to an excess of intermediate frequency mutations (see below). There is a greater effect on π as long as the two subsets show similar sizes (this reversed pattern is indeed not detected in situations where the ancient subset represents 10% or 90% of the overall sample thereby leading to fewer intermediate frequency mutations; Fig. S4).

The overestimation of polymorphism indices can be easily explained, for instance, for the pairwise diversity estimate *π* [42], which can be thought as the two sequence sample case. If a pair of sequences shows time spacing, their lineages cannot reach a common ancestor before they are contemporaneous and once they are contemporaneous, their additional coalescent time is the same as that in the contemporaneous case (Fig. 1B vs 1A). The total time to their common ancestor is then increased and there is an additional branch fragment on which mutation can occur, thus inflating the pairwise diversity *π*, the number of polymorphic sites, *S*, and consequently *θ_W_* (proportional to *S*; see Forsberg, Drummond and Hein [55] and Liu and Fu [56] for more general analytical results).

A corrected diversity index providing an unbiased estimator of θ corrected for heterochrony is then given by
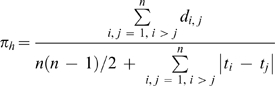
(1)where *d_i,j_* is the number of differences between sequences *i* and *j*, and *t_i_*–*t_j_* the time lapse between them, with times expressed in 2*N_e_* generation units (see circularity issue discussed in text S1, simulation subsection). The term on the right side in the denominator is correcting for heterochrony.

If estimates of the generation time and of the mutation rate (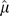) are available (e.g., from phylogenetics with fossil scaling, pedigree studies), a corrected diversity is then provided by
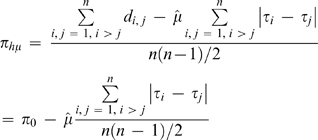
(2)where *π*_0_ is the contemporaneous diversity estimator and the *τ* times are expressed in generations.

### Neutrality tests and genealogy topology

Temporal spacing inferior to 2*N_e_* generations leads to star-like trees (Fig. 1B, 3, 4) and an excess of rare mutations, as reflected by the negative Fu and Li's, Tajima's frequency statistics, and a deficit of haplotype structure (increased *K* and *H*) and pairwise linkage disequilibrium, *Z_nS_*; Fig. 3A and 4A). This star pattern is a typical (spurious) signal of for instance population expansion, possibly after a strong bottleneck. In contrast, and not unexpectedly, other statistics remain virtually unaffected. The topology of a genealogy, e.g. its symmetry (reflected by *H_FW_*), is indeed not depending on the age of the tips (in contrast with the length of the branches, see comment to Fig. S4 below). Similarly, under the IMSM and in absence of recombination, the position of mutations along the sequence is not informative. Thus, no relation is expected between the distance between polymorphic sites and LD, as reflected by unaffected Pearson recombination test (“slope”).

For the cases corresponding to (virtually) no temporal spacing (rightmost and leftmost points on figure 2), the proportion of significant runs for all tests was, as expected, close to the chosen nominal value (5% and thus not apparent on figure 3B). For parameter values maximizing the average time spacing between pairs of sequences (*n*_1_ = *n*/2), about twice as many runs were significant (e.g., up to 12% for D*_FL_, 11% for *Z_nS_* and 10% for *D_T_* tests, Fig. 3B).The effect decreased roughly symmetrically around this subset size of half the total sample.

On the whole, these results indicate substantial non-robustness of the tests (too many false positive) with respect to heterochrony.The counterpart is that the capacity (the power) of the test to detect opposite scenarios such as simple geographical genetic isolation should be correspondingly decreased.

Similarly, on figure 4, left hand side, for low or null time lapse (*t*_1_), there is no excess of false positive tests (5%). But for moderate time lapse of the subset (0.05–2 *N_e_* generations, i.e. 0.1<*t*_1_<1), the proportion of significant runs reached values as high as three times this expected threshold. This covers a typically relevant range of values, in particular for aDNA data, which are limited to the last tens to hundreds of thousand years due to the kinetics of the taphonomic DNA degradation process [57], [58]. For greater time lapse above 2*N_e_*, probably more relevant for viral evolution, the effect is generally drastic: the most recent subset generally reaches its root before the ancient one is included in the tree, thus leading to a balanced tree with long internal branches. With two subsets of comparable sizes as in figures 1C and 4, this balanced pattern is reflected by an excess of intermediate frequency mutations and departure of the tests in the opposite direction, thus mimicking e.g. simple population substructure (stratification) in the total sample. However, when the two subset sizes differ substantially, this opposite direction effect for large time spacing is lessened (Fig. S4). When most of the sample is recent (*n*_1_/*n* = 90%), an excess of high frequency derived mutations is observed (those occurring on the long internal branch leading to the modern subset, the two additional stripped ones on figure 1C), resulting in a spurious signature of positive selection (negative *H_FW_*).

### Population structure

The total sample was artificially randomly split into two population subsamples of equal sizes (*n_A_* = *n_B_* = *n*/2) as if they came from different geographical location though all the simulations involved a single well mixed population (without any limitation of flow between the subsamples). The *F_st_* was computed between the two population subsamples. On all of the simulation outcomes, the *F_st_* was tested by permutations of sequences between the two population subsamples. Studying extensively the combination of temporal and geographic sampling schemes would be cumbersome. We rather focus on extreme representative simple cases.

On figure 3 , the *X* axis represents the proportion of the second population coming from time *t*_1_ (as outlined on Fig. 2B) whereas on figure 4, the whole second population sample comes from time *t*_1_ (*X* axis, *n_B_* = *n*_1_ = *n*/2) while the first one is entirely recent (*t* = 0, *n_A_* = *n*_0_ = *n*/2). Figure 4 then represents the worst case scenario where the two populations have samples from different times (note that this fits most aDNA studies, as different excavation sites are typically associated with different stratigraphic layers and as for non-extinct species, ancient haplotypes are most generally compared to extant ones; see for instance studies on brown bears [12], [18], [19] or bisons [36]). The proportion of significant runs increased continuously with the time spacing *t*_1_ (Fig. 4). This can rapidly lead to substantial *F_st_* values (0.09 for *t*_1_ = 0.1 and 0.91 for *t*_1_ = 10) and great power (73% and 100%, respectively). On figure 3 (*t*_1_ = 0.1), the *F_st_* increases regularly as a function of the proportion of ancient sequence in the second subset (0.001 for *n*_1_/*n_B_* = 0.1 and 0.085 for *n*_1_/*n_B_* = 1) with dramatically increasing power (5% and 72%). These results indicate that -possibly strong and yet- spurious population structure signal can potentially result from different serial sampling schemes in the two population subsamples (in agreement with [38]). Similarly if there is real (geographical) population structure in the data, heterochrony can only strengthen it so that the level of isolation (or the time since an ancestral population splitting in isolation models) would be overestimated. Conversely, in models involving limited migration such as island models, the associated estimates of gene flow should be underestimated. Perhaps more interestingly if population subsamples show similar time sampling schemes involving some heterochrony, the average level of differentiation would not be affected but the power of the associated test (the efficacy to detect significant differentiation) would be reduced because the distribution of the statistics would be more widespread (in particular, the variance would be inflated).

We therefore propose a corrected estimator of the distance between populations (similar to the one introduced for correcting diversity estimates):
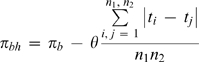
(3)where *π_b_* is the uncorrected estimate and θ can be replaced by its estimate from equations (1) and (2) (with some average of within population estimates), or when generation time and mutation rate estimates are available,
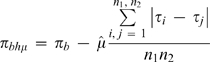
(4)in generation units.

### Sampling schemes

**Figure 5:**
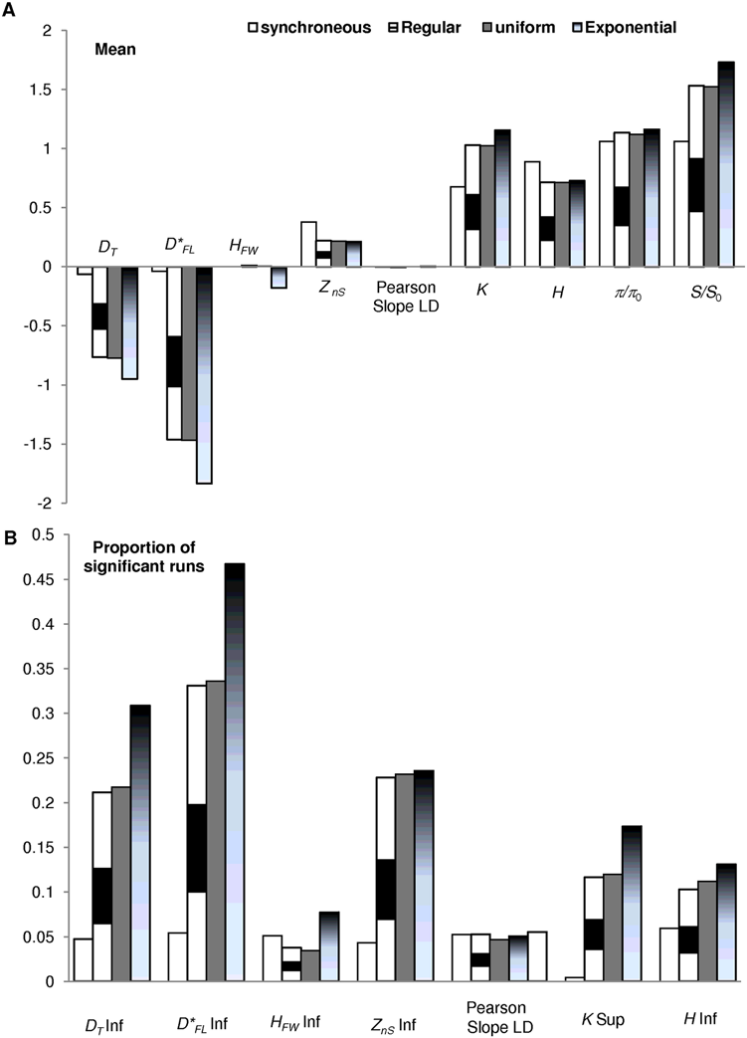
Effect of time sampling schemes on the statistics. (a) Means. For comparison, statistics with non-null means in the contemporaneous case are scaled to the upper bound of their confidence interval under such null hypothesis. (b) Proportions of significant runs only in the direction of deviation potentially leading to deviation (if any) in the heterochroneous case are shown (the other one remaining below 5%). ‘inf’: deficit of the statistic; ‘sup’: excess of the statistic. Open bars: contemporaneous; stripped bars: regular in the range [0–0.2]; homogeneous gray bars: uniform, same range; gradient-filled bars: exponential with mean 0.1 (truncated at 10 to limit CPU and assuming that there was no chance at all to obtain as old DNA for a species that may not have even existed at that time).

Moving from the simple two population case, we modeled different extreme time sampling scenarios from individuals belonging to a single global population (Fig. 5): (*i*) contemporaneous samples (i.e., assuming no heterochrony), (*ii*) regular, (*iii*) uniform and (*iv*) exponential sampling across time. Cases (*ii*), (*iii*) and (*iv*) are set for equivalent average *t*_1_ (0.1). Case (*i*) meets the typical modern day samples or ancient samples with all of the sequences derived from the same time (coming from the same stratigraphic layer *t*_1_ = 0.1), (*ii*) represents studies trying to meet an ideal case with the most exhaustive time sampling scheme on a given time range (0–0.2×2*N_e_* generations), with good DNA preservation conditions over all of the stratigraphic layers, (*iii*) is just random sampling among layers assuming that aDNA would get the same chance to be recovered whatever the age of the fossils within a time range (0–0.2×2*N_e_* generations); (*iv*) represents a case where aDNA would suffer a constant rate of degradation (average *t*_1_ = 0.1×2 *N_e_* generations). This analysis can also be viewed as extreme distributions for modeling uncertainty in time estimates.

Again, heterochrony leads to an excess of rare variants providing spurious support for star scenarios (Fig. 5; negative Tajima's *D* and Fu and Li's D*, a deficit of linkage disequilibrium), with virtually no effect on tree symmetry (*H_FW_*) or the Pearson recombination test. The difference between the sampling strategies is weak; the average *t*_1_ primarily matters, though the exponential distribution shows the greatest effect primarily because the distribution of times is more widespread with such sampling schemes with long tails (truncation in *t*_1_ = 0.2 like other time sampling schemes provides similar results for the three distributions; results not shown). Strikingly, the proportion of false positive runs due to time structure is substantial ranging from 10 to 50% depending on the sampling scheme and the statistics. Importantly, the most affected ones are the most classic ones (Fu and Li's D*, Tajima's *D*, plus *Z_nS_* the latter being most independent of the sampling scheme).

### Application to the Cave Bear data

We applied our procedure to the Cave Bear ancient DNA dataset. This represents a more complex time sampling scheme than in the examples presented above (Tables 2 and 3 and Fig. S1) and time lapse ranging up to 20% of the estimated age of the MRCA, though the oldest times (in the range of 80–130 KYA) are being represented by only 12 sequences. In addition to the total dataset – which may be geographically structured and includes 44 ancient sequences of unknown age (or roughly estimated due to radiocarbon limits) – we also applied the analyses on all local populations with more than four individual sequences and three segregating sites (and, for the *F_st_* analyses, between all pairs of population or between the sets of populations on each side of the Alps).

**Table 2:** Polymorphism estimates from caves of Cave Bear.

Cave	*n*	*S*	Time range (KY)	Average pairwise time difference (KY)	*θ_W_*	*π*/pb	*π_h_*/pb^a^	%bias
Ach	20	13	25–39	3.3	0.028	0.047	0.047	0.7
Herdengel	8	10	55–130	10.7	0.030	0.030	0.030	4.6
Scladina	20	15	30–130	36.6	0.052	0.040	0.037	8.7
S Alps	22	9	22–130	22.7	0.036	0.017	0.016	8.2
N Alps	33	21	22–130	32.9	0.039	0.047	0.046	3.0
Total	118	19	22–130	31.3	0.052	0.068	0.063	8.7

a Corrected from equation (1).

**Table 3:** Neutrality tests on Cave Bears^a^.

Cave	Time range (APTD)^b^	*T* c	*D_T_*	*P* c	D*_FL_	*P*	*H_FW_*	*P*	*Z_nS_*	*P* d	*r* e	*P*	*K*	*P* d	*H*	*P* d
		*c*		0^**^		4^*^		24		1^**^_+_		23		11_−_		23_−_
Ach	25–39 (3.3)	*h*	2.60	0^**^	1.48	3^*^	−0.67	23	0.79	0^**^_+_	−0.05	23	5	9_−_	0.72	22_−_
		*hu*		0^**^		3^*^		24		1^**^_+_		22		9_−_		22_−_
		*c*		14		4^*^		31		23_+_		55		33_−_		39_−_
Herdengel	55–130 (10.7)	*h*	0.96	11	1.50	3^*^	1.50	34	0.51	18_+_	0.06	53	4	25_−_	0.72	32_−_
		*hu*		9		2^*^		35		15_+_		52		19_−_		27_−_
		*c*		22		10		20		43_+_		4_*_		35_−_		41_−_
Scladina	30–130 (36.6)	*h*	−0.82	35	−1.55	18	−1.32	17	0.24	27_+_	−0.39	5_*_	7	16_−_	0.79	30_−_
		*hu*		41		24		20		23_+_		4_*_		12_−_		29_−_
		*c*		2^*^		4^*^		1^**^		3^*^_+_				8_−_		2^*^_−_
S Alps	22–130 (22.7)	*h*	−1.75	4^*^	−2.35	6	−5.84	1^**^	0.63	1^*^_+_	/		4	4^*^_−_	0.38	1^**^_−_
		*hu*		6		9		1^**^		1^*^_+_				3^*^_−_		1^**^_−_
		*c*		15		18		17		37_+_		77		0^**^_+_		1^*^_+_
N Alps	22–130 (32.9)	*h*	0.86	4^*^	−1.17	58	1.83	6	0.14	14_+_	0.08	77	22	0^**^_+_	0.89	1^**^_+_
		*hu*		2^*^		39		4^*^		11_+_		77		0^**^_+_		0^**^_+_
		*c*		10		29		8		31_+_		81		0^**^_+_		5_+_
Total	22–130 (31.3)	*h*	1.12	2^*^	−0.80	42	2.05	2^*^	0.15	11_+_	0.11	81	23	0^**^_+_	0.87	3^*^_+_
		*hu*		1^**^		23		1^**^		7_+_		80		0^**^_+_		2^*^_+_

a All simulations are conditioned on the observed number of variable sites (*S* value). For each statistic, the observed value is indicated on the left.

b Time range and Average pairwise time difference in KY.

c On the right of each statistic, probability (%) on 3 lines (corresponding to the legend in the *T* column): first line, ‘*c*’ assuming contemporaneous sample; second line, ‘*h*’ taking into account heterochrony, with an average time for each sequence; third line, ‘*hu*’ also including time and parameter uncertainty with uniform deviates (ranges detailed in figure S2).‘*’: *P*<0.05; ‘**’ *P*<0.01.

d The direction of deviation is indicated when not obvious (the contemporaneous expectation for frequency spectrum and Pearson statistics is 0): ‘_+_’ excess, ‘_−_’ deficit.

e Pearson correlation between LD and distance test, permutation test.

We estimated the level of polymorphism and assessed whether there was any departure from the neutral model and any evidence for geographical population structure, as this could be indicative of bottlenecks and limited gene flow or long-term isolation between populations, respectively. Such intrinsic factors may have contributed to the extinction of the species. Gene flow in particular is essential for the recolonization of extinct local populations and polymorphism tends to favor for sustainability by overcoming inbreeding depression and providing the basis for adaptation to environmental changes. Interestingly, studying such features on extinct species offers a unique opportunity to address conservation issues. We applied all tests with and without modeling the heterochrony effect to illustrate how sensitive to heterochrony the conclusions are.

For all alignments, we performed three sets of analyses: *(i)* assuming contemporaneous data (Table 3, “*c*” lines); *(ii)* taking into account heterochrony with a single average estimated age for each sequence (“*h*”) and, as time estimates generally show large uncertainty, *(iii)* considering the uncertainty (uniformly distributed within a given range; “*hu*”) in the estimated age and in the parameter values (*N_e_* and generation time). For the total sample analyses, in the absence of a clear prior, the sequences without any time information were assigned an average time within the range of times available for other sequences (22–130 KYA, we used an unweighted average: 76.0 KYA, which is close to the weighted one: 59.6 KYA). Note that the dataset is hardly “measurably evolving” (*sensu* [5]): for instance, on a haplotype network, the correlation between the distance (number of mutational steps) from the most parsimonious ancestral sequence and the time to the sequences, though significant at the 5% level, explains only 4% of variation (see Fig. S4).

### Cave Bears: intrapopulation analyses

Polymorphism estimates ranged from more than a factor of four among polymorphic populations beyond population samples not analyzed as showing virtually no information (Table 2). Diversity estimates were corrected for heterochrony with equation (1). Importantly, depending on the subsample and the way to partition the data, heterochrony leads to up to 9% overestimation of polymorphism level when the subsample showed a substantial time range and average time difference between pairs of sequences (compare *π*/pb to *π_h_*/pb columns; their standardized difference is summarized in the % bias column). This leads to artificially underestimate heterochroneous population extinction risks.

Neutrality test conclusions were affected unevenly by time structure (Table 3, *P* values columns). For limited time ranges or average pairwise time difference and far away from the acceptance threshold, heterochrony does not affect the conclusion of many tests. However, some tests reached the significance level when heterochrony and (or) time uncertainties were taken into account. For instance the total and North of the Alps datasets showed positive *D_T_* (e.g. population stratification) and *H_FW_*, and there was a deficit of haplotypes *K* in the south of the Alps, which turned significant when considering heterochony. Similarly, correcting for heterochrony strengthened some significance levels such as the *H* test on the south (deficit) and the north (excess) of the Alps. Conversely, some tests passed below the significance level when modeling heterochrony (e.g. negative *D_T_* and D*_FL_ on the south of the Alps; Table 3), thereby removing any evidence for non standard evolution (such as demographic expansion; Table 3). In general, the standard null model appears less likely when heterochrony is taken into account, thereby suggesting that accurate modeling increases the power of the tests to detect deviations from standard scenarios (e.g., demographic changes; results consistent with [32]).

On the whole, as to the history of Cave Bears, a few congruent points seem to pertain: the patterns observed in Ach valley, in the north of the Alps and in the total samples are consistent with a strong geographical population structure within the dataset (stratification) including several distant populations with substantial sample sizes [47]: positive frequency statistics, excess of linkage disequilibrium (see next section). The excess of haplotype and haplotype diversity are likely to be due to the presence of homoplasies (recurrent mutations) in addition to time spacing (the number of haplotypes exceeds *S*+1, the maximum possible value in the absence of homoplasy or recombination). Similarly, the Fay and Wu neutrality test [49] has already been shown to be highly sensitive to homoplasies [59] which may contribute to the significantly negative value South of the Alps (see related point in the discussion). A closer look at the raw data suggests that the observed pattern in this latter subsample (negative frequency statistics excess of linkage disequilibrium, deficit of haplotypes) seems to result from population stratification with a distant population (Conturines) represented by a single (distant) sequence, which may reflect a separated refuge zone from the previous glaciations era: Italian versus Balkan. Interestingly, in the Belgian (Scladina) subsample, we found a significant negative correlation between the pairwise linkage disequilibrium and the distance between mutations along the sequence (‘Pearson's test’, *r* = −0.45 , *P* = 4%; Table 3, Fig. S5). The power of this test should however be low given the small number of informative (non unique) mutations (six sites in Scladina). This test was not affected by heterochrony.

### Cave Bears: population structure

Most populations appear highly differentiated (Table 4). The permutation tests are generally significant whenever there is substantial information (more than four sequences per population and three segregating sites). However, the evidence for genetic isolation (the level of significance) is often weakened (e.g. see the Austrian populations from Ramesch and Winden) or disappears (e.g. see the differentiation between the Austrian and Belgian populations from Salzofen and Scladina) when heterochrony and (or) time uncertainty are taken into account (greater *P* values) especially when the time ranges of the two populations is widespread (or the average pairwise time difference is large; e.g. comparisons involving Herdengel, Ramesch Salzofen and Scladina). The correction of distances with equation (3) was sufficient to change the topology of phylogeny between populations (with most simple and direct tree reconstruction method, an unweighted pair group method with arithmetic mean: UPGMA; Fig. S6).

**Table 4:** Population differentiation between caves of Cave Bears^a^.

	Ach recent	Ach old	Gamsulzen	Herdengel	Ramesh	Salzofen	Scladina	Vindija	Winden	S Alps	N Alps
Time range (KY)	25–28	27–39	31–50	55–130	30–130	22–130	30–130	22–51	22–130	22–130	22–130
Cave \ *n*	(7)	(13)	(7)	(8)	(9)	(4)	(20[Table-fn nt108] b/6[Table-fn nt108] c)	(12)	(7)	(22)	(33)
		(15)	(3)	(12)	(12)	(12)	(15)[Table-fn nt108] b	(4)	(1)		
Ach recent		0.083	0.015	0.041	0.089	0.089	0.080	0.007	0.007		
		0.083	0.014	0.037	0.086	0.084	0.080	0.006	0.003		
	0^**^		(12)	(16)	(12)	(7)	(14)[Table-fn nt108] b	(13)	(12)		
Ach old	0^**^		0.064	0.028	0.031	0.024	0.002	0.069	0.076		
	0^**^		0.063	0.025	0.028	0.029	0.002	0.069	0.072		
	2.18										
	0^**^	0^**^		(11)	(11)	(11)	(19)[Table-fn nt108] c	(3)	(2)		
Gamsulzen	0^**^	0^**^		0.024	0.068	0.065	0.057	0.000	0.007		
	0^**^	0^**^		0.021	0.066	0.062	0.057	0.000	0.003		
	1.87	2.93									
	0^**^	0^**^	0.1^**^		(10)	(10)	(20)[Table-fn nt108] c	(12)	(11)		
Herdengel	0^**^	0^**^	1.7^*^		0.014	0.001	0.014	0.033	0.033		
	0^**^	0^**^	1.4^*^		0.012	0.001	0.013	0.030	0.033		
	7.90	5.37	6.27								
	0^**^	0^**^	0^**^	0^**^		(0)	(14)[Table-fn nt108] c	(12)	(16)		
Ramesh	0^**^	0^**^	0^**^	0^**^		0	0.035	0.071	0.082		
	0^**^	0^**^	0^**^	0^**^		0	0.033	0.069	0.079		
	6.16	3.78	4.83	2.17							
	0^**^	0^**^	0.2^**^	29.4	/		(4)[Table-fn nt108] c	(12)	(11)		
Salzofen	4.0^*^	0^**^	0.2^**^	27.4	/		0.017	0.068	0.082		
	3.0^*^	0^**^	1.0^**^	41.2	/		0.016	0.065	0.082		
	9.84	8.82	10.43	12.19	10.79						
	0^**^	23	0.2^**^	0.3^**^	0^**^	1.1^*^		(20)[Table-fn nt108] c	(19)[Table-fn nt108] c		
Scladina	0^**^	59	0.2^**^	2.4^*^	0^**^	3.6^*^		0.069	0.072		
	0^**^	55	0.4^**^	3.0^*^	0^**^	5.7		0.069	0.069		
	9.64	4.43	9.56	9.95	8.20	19.52					
	0^**^	0^**^	26.4	0^**^	0^**^	0.0^**^	0^**^		(3)		
Vindija	0^**^	0^**^	27.8	0^**^	0^**^	1.4^*^	0^**^		0		
	0^**^	0^**^	29.7	0^**^	0^**^	0.9^**^	0^**^		0		
	3.30	0.91	3.26	5.39	3.71	9.19	4.72				
	0^**^	0^**^	0^**^	0.1^**^	0.1^**^	0.2^**^	0^**^	/			
Winden	0^**^	0^**^	0^**^	0.2^**^	0.3^**^	0.2^**^	0^**^	/			
	0^**^	0^**^	0^**^	1.7^*^	2.2^*^	0.9^**^	0^**^	/			
	7.09	5.71	5.16	2.89	3.58	8.14	11.28				
											(19)
S Alps											0.015
											0.018
										0^**^	
N Alps										0^**^	
										0^**^	
										2.70	

a Top right: line 1, in parentheses, number of polymorphic sites in the pairwise alignment; lines 2 and 3, Nei's net distances *D_a_*, line 2 uncorrected; line 3 corrected for heterochrony with equations (1) and (3). Bottom left: *P* values from permutation tests, significance level; line 1 neglecting heterochrony; line 2 taking it into account; line 3 including uncertainty; line 4: Inter-population average pairwise time difference (KY). The number of sequences used for each population is given in parentheses at the top of the columns.

b, c Variable number of sequences depending on the alignment chosen to maximize information.

b *n* = 20.

c *n* = 6.

Interestingly, the different caves in Ach valley sampled from different times are highly differentiated, as described in [26] and heterochrony alone is not sufficient to explain this pattern. These caves do not share haplotype groups, but it is not clear whether this pattern is due to (1) small scale geographic isolation or (2) temporal structure combined with gene flow such as colonization processes (as interpreted by Hofreiter and colleagues, the “haplotype replacement” hypothesis) since haplotypes from the different caves are not contemporaneous. Finally, the northern and southern sides of the Alps were highly differentiated (despite more local geographical structure within each side) suggesting a geographical barrier to gene flow across the Alps. Such a limited gene flow (and associated inbreeding depression) may have contributed to the extinction of the species 12–20 thousand years ago.

## Discussion

### Coalescent model concerns: heterochrony-driven systematic biases

Our simulation results indicate that population genetics analyses can be substantially biased by heterochrony in a dataset. What matters is a balance between generation time, effective and sample size (the rate of common ancestry: probability to reach a common ancestor per time unit) and time spacing: the greater the time spacing and the sample size, and the smaller the generation time and the effective size, the greater the heterochrony effect.

**Figure 6:**
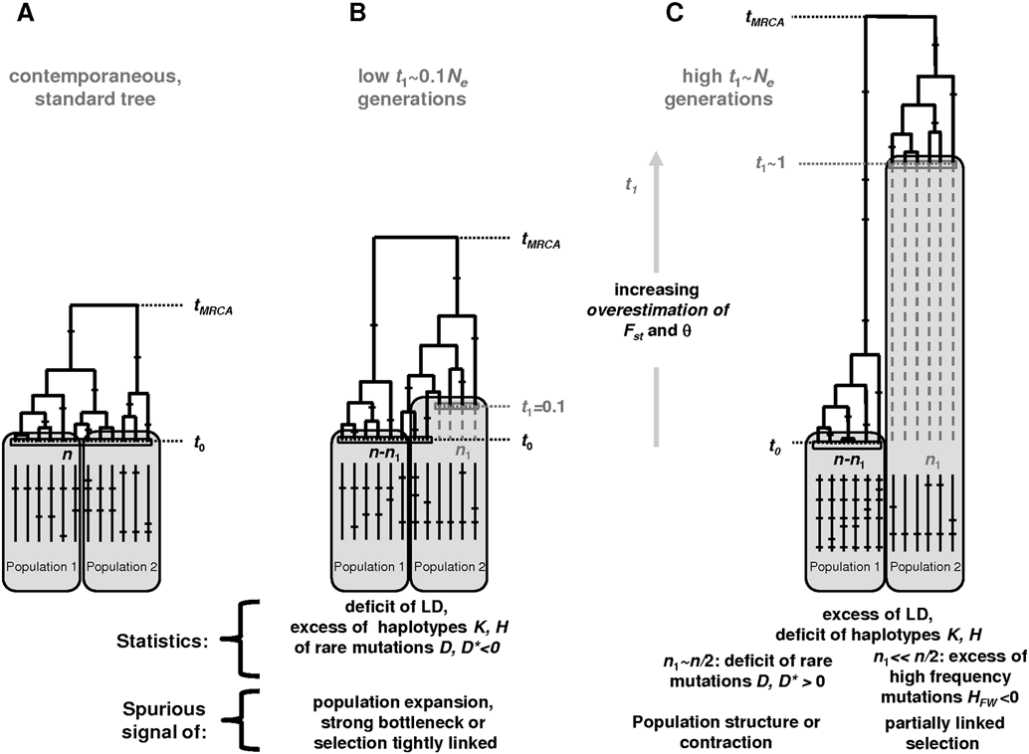
Heterochrony-driven biases on summary statistics: a synthesis. (A): contemporaneous case. (B) Heterochroneous dataset with limited time range. Lineages of sequences cannot reach a common ancestor before they are contemporaneous, leading to genealogies with proportionally longer external branches and excess of rare mutations thus mimicking bottlenecks, expansions or tightly linked selection. (c) Two subsets separated by a large time lapse. The coalescence process is finished within the most recent subset before reaching the ancient subset sampling point, leading to a genealogy with a long internal branch, more variation, especially for intermediate frequency mutations, and a genetically isolated subset, thus mimicking simple population structure or contraction. *t*_1_: time lapse; *n*_1_: oldest subset's size.

For most relevant sets of parameter values compatible with most classical aDNA analyses, i.e., limited time lapse with respect to the root of an intraspecific tree, heterochrony leads to a shift toward star-like trees, revealed by negative frequency statistics and a deficit of association between mutations, thus mimicking population expansion possibly following a drastic bottleneck (Fig. 6A). Sequences cannot coalesce before they are contemporaneous, and thus branch lengths tend to increase (Fig. 6 A vs 6B). External branches tend to be proportionally more affected. First they are more numerous than internal ones, second, they always include the tips of the tree involved in the time spacing, and last, they tend to be shorter. Greater heterochrony (on the order the depth of an intraspecific tree) generally causes the most recent subset to coalesce before the ancient one is added to the tree, thus leading to a long internal branch splitting the set of sequences between the ancient and the more recent subsets and balanced like trees with long internal branches (Fig. 6C). When the subsets are well balanced, this results in departures of the statistics in the opposite direction, with potentially strong effects mimicking simple cases of population isolation. In addition, polymorphism and distance estimates are then substantially increased. Such large heterochrony could be appropriate for microbial evolution, but is not necessarily out of the range of aDNA, even for cave bears as 300 KY old authentic genetic data has recently been reported [60].

A practical consequence is that it is not possible to compare directly values coming from different time sampling schemes (and in particular modern vs ancient heterochroneous data). Rather, corrected equations (1–4) or an explicit serial modeling [19] should be used. Not doing so would tend to overestimate the level of polymorphism of the ancient population, which may in turn support fake demographic declines (as the level of polymorphism tend to correlate with population sizes) and promote inappropriate conservation decisions, considering that genetic variation provides the material for adaptation to environmental shifts.

For simple time sampling schemes, with only two different sampling times in the dataset, the heterochrony effect is the strongest when the two subsets were of comparable sizes, and this effect is roughly symmetrical around this value. The effects are substantial even when the temporal heterochrony is limited to a range of values less than 0.2 *N_e_* generations (<10% of the average age of the depth of a standard tree). This case, probably the most relevant in practice for ancient DNA, leads to double the proportion of significant runs in neutrality tests. In this case heterochrony leads to spurious signal of star scenarios such as population expansion possibly after a strong bottleneck, the recent spread of a tightly linked advantageous mutation, or complex population structure schemes (e.g. a collection of small samples from a substantial number of isolated populations). In contrast two subsets of similar sizes separated by greater time spacing (on the order of the depth of a typical tree) can lead to spurious balanced tree generally interpreted as signals of population contraction, moderate bottleneck or simple population isolation between those subsets.

With a real and more complex heterochronic structure, such as that shown by the Cave Bear dataset, the heterochrony effect was sufficient to change the conclusions of several tests applied to the dataset. On the whole, the neutral model showed a poorer fit when taking into account the heterochrony. Once corrected for heterochrony, strong signal of population structure remain, even on a limited geographic scale. Such limited gene flow, recolonization capabilities, may have contributed to the extinction of the species.

Another important concern is related to uncertainty in time estimates, which may generally be large, especially for aDNA and when including other parameter uncertainty (such as for *N_e_*, or the generation time). We observed a rather weak effect of this uncertainty on the analyses (Fig. 4), especially when reasonable time information is available. Most of the effects that we observe seem to arise from the average heterochrony and not from the time estimation uncertainty.

Orlando and colleagues [28] noticed shifts in average pairwise genetic diversity between three stratigraphic layers (within one cave bear haplogroup). These shifts seemed synchronous with shifts in global environmental conditions (glacial or interglacial), which suggested possible ecological interpretations about demographic dynamics. Here, we showed that heterochrony within a dataset could, to some extent, increase the diversity index. However, the results observed seem unlikely to be an artifact of the heterochrony within layers since subsets with similar ranges in heterochrony (Fig. 3 of [28]) show a high difference in pairwise genetic diversity (80–120 and 90–130KYA, Fig. S3). [Note, however, that none of the differences are significant given the large sampling and stochastic variances and the non-independence between the various pairs of sequences involved in the comparisons, suggesting that more data from supplemental individuals and independent loci are required before reaching a conclusive level.]

### Mutational model concerns

Interestingly, our Belgian Cave Bear mitochondrial DNA dataset show a significant negative correlations between the linkage disequilibrium and the distance between the mutations (Table 3: ‘Pearson's test’, *r* = −0.39, *P* = 4%; Fig. S5). Note that the Scladina cave in Belgium is the most relevant subsample to test for this correlation, as it is entirely derived from a local population, thus minimizing population structure effects on linkage disequilibrium measures (which are known to be drastic [61]).

Such correlations have generally been taken as evidence for recombination, including for human mitochondria [53], [62]. This view was however strongly debated with criticisms about the quality of the data and about the measure of linkage disequilibrium used [63]–[67]. [Note that most criticisms did not explain or predict the observed correlation.] The observed correlation of Cave Bears fits an exponential relationship, as approximately expected with recombination effects (Fig. S5). Here however, recombination seems rather unlikely, since, given the short length of the aligned region (81 bp), recombination rates orders of magnitude higher than the autosomal rates would be needed. We also showed that this pattern could not result from heterochrony. Consequently, mutational effects similar to those described by Innan and Nordborg [68] with mutation hot spots in one region, which tend to reduce short distance linkage disequilibrium, seem a more likely explanation. Similarly, clumping of mutations along the sequence and across the genetic history of the population or complex mutational events, substituting simultaneously several neighboring nucleotides, can lead to such apparent signature of recombination, even in the absence of multiple hits or homoplasies on the same site (F. Depaulis; unpublished results). At any rate, mitochondrial DNA do not obey the assumptions of the infinitely many site mutational model (IMSM; [69]). This is particularly true for the control region, which shows strong heterogeneity of mutational rates [70] and high transitional biases [71], [72]. Note, however, that in [53] the control region was removed from the analysis and that the other sites did not show apparent multiple hits. There is no direct evidence for multiple hits on the Cave Bear dataset (no site with more than two nucleotide variants), which is probably due to the high transition transversion bias thereby making such multiple mutations on a site not readily apparent. However, apparent homoplasies (or recombination events) are detected between several pairs of polymorphic sites (four gamete rule analysis [73], *R_m_* = 5 on the total dataset; the same –extended- principle states that the number of haplotypes cannot exceed *S*+1 in the absence of such events, see intrapopulation cave bear results and table 3).

More generally, the departure from the IMSM due to homoplasies is a major concern, especially in ancient DNA analyses where most data still rely on the mitochondrial hypervariable region and for viruses generally showing high mutation rates. Indeed mutational effects (as well as heterochrony) are relevant alternative hypotheses when faced with significant neutrality tests. For instance, the excess of haplotypes and haplotype diversity found on the total Cave Bear dataset should largely result from homoplasies. This may also contribute to the significantly negative Fay and Wu neutrality test in the south of the Alps. In the presence of homoplasies, most undetected mutations would occur in the deep part of the tree and consequently should not be affected by heterochrony. The practical consequence is that, when not taking into account such mutational effects, the time uncertainty effect is underestimated.

Similarly, most recently Axelsson and colleagues [74] reported that DNA damage in aDNA data can largely affect demogenetic inferences. Such noise in the data should drastically enhance the effect we describe here since it also tends to lengthen the external branches of genealogies. An additional caveat for aDNA is that data are not necessarily sequenced on the same sequence fragment for all individuals so that there are a number of missing data in the whole data alignment. Similarly, sequencing error, usually magnified for aDNA due to chemical damage, could also be treated as missing *biological* data for the analyses to focus on relevant information. In such cases, summary statistics (and associated tests) will not be technically straightforward to compute as the sample size may vary from site to site in a non independent way. Consequently, adequate methods should be urgently developed.

In view of the above results, it seems necessary to systematically take heterochrony into account for most heterochronous dataset analyses even if the time range seems rather limited (around 10%) with respect to the age of the MRCA.

## Supporting Information

Text S1 Supplementary methods, alternative approaches, simulations and associated references
(0.16 MB PDF)

Figure S1 Algorithm for simulations of heterochroneous genealogies. The classical algorithm (exponential coalescent times) starts with the most recent subset (A); until a coalescent time exceeds the time to the next subset *t*_1_; then (B) the event is cancelled and the algorithm starts back from time *t*_1_ with a number of lineages *g* updated by adding *n*_1_, until (C) the MRCA is reached.
(0.08 MB PDF)

Figure S2 Effect of heterochrony on statistical tests as a function of time spacing. (A, B): 10% subset;(C, D) 90% subset (*n*_1_ = 10 or 90, respectively, whole second population subsample in the *F_st_* analysis). The *X* axis is expressed in units of 2*N_e_* generations. Same labeling as in figure 2. The effects of other parameters such as total number of sequences in the dataset and polymorphism levels were investigated elsewhere [S18] and do not show noticeable interaction with the heterochrony effect (the effects described here simply appear stronger for larger datasets especially for increasing sample size since when the heterochrony range is limited and affects mostly the short external branches.
(0.18 MB PDF)

Figure S3 Alignment of polymorphic sites of the Cave Bear dataset. For each site, the given number refers to the position relative to the first nucleotide of the sequence under Accession Number AY149238. Accession numbers are reported as sequence names. For haplotypes that stem from different non-overlapping sequences, a list of corresponding Accession numbers is given below the alignment. Haplotypes are referenced according to the following: AccessionNumberIfAvailable_Name_MinimumAge_MaximumAge_(Location.
(0.13 MB PDF)

Figure S4 Median-joining Haplotype network [S19] of the Cave Bear dataset. The sizes of the nodes are proportional to their frequencies. Each location is indicated by different colors. The most parsimonious ancestral state (“reference” in figure S1) is boxed in green. The average (or minimum whenever the average could not be computed) time to the sequenced is boxed in grey near the nodes. Correlation between the average age and the minimum number of mutational steps from the ancestral state: *r* ^2^ = 0.04*. The correlation between the age of the sequences and the genetic distance from the most parsimonious ancestral state is hardly significant, suggesting that there was too little information to estimate a whole set of parameters reliably under a full MCMC likelihood framework (see text S1). Indeed when we tried to apply the likelihood method of Drummond and colleagues [S7] to the data it was not able to disentangle the effective size from the mutation rate (highly correlated posterior distribution and we needed to provide an independent estimate of the mutation rate to estimate the effective size properly. We therefore did not rely on such approaches to assess the heterochrony driven bias.
(0.12 MB PDF)

Figure S5 Allelic correlation (*r* ^2^) as a function of distance between informative sites in the Belgian Cave Bear subsample. The *Y* axis is on a log scale as an exponential relationship is approximately expected for the recombination effect (strictly, this corresponds to the expectation under a deterministic approximation). An exponential regression is shown for comparison.
(0.06 MB PDF)

Figure S6 UPGMA tree between Cave Bear populations from pairwise distances. A: uncorrected. B: corrected for heterochrony.
(0.06 MB PDF)

Table S1 List of Accession Numbers for sequences that stem from different non-overlapping PCR fragments. Some samples could be associated with an identical Accession number as they have been reported to exhibit identical haplotypes.
(0.14 MB PDF)
