# Supplementary material for: Using Classical Population Genetics Tools with Heterochroneous Data: Time Matters!
Source: PLoS One. 2009 May 14;4(5):e5541. doi: 10.1371/journal.pone.0005541 (PMC2678253; doi:10.1371/journal.pone.0005541)
Supplement: Text S1 — Supplementary methods, alternative approaches, simulations and associated references (0.16 MB PDF) [file pone.0005541.s001.pdf]

Introduction to alternative approaches:

Analytical results for identity by descent coefficients in heterochroneous data have been derived under a spatial context [S1]. These coefficients have been used to derive coalescent times [S2]. Other authors [S3-S5] derived some statistical properties of trees (e.g., the time to the most recent common ancestor -MRCA- the expected number of segregating sites, shared or not between ancient and modern sequences) under a simple Wright-Fisher, infinitely many site mutational model (IMSM) [S6]. Liu and Fu [S5] also derived some summary statistics related to the F_{st} (standardized Nei distance) to test for heterochrony.

In addition to analytical statistics, simulation-based methods considering heterochrony have been proposed under likelihood (generally Bayesian) Markov chain Monte Carlo (MCMC) framework including mutational and demographic models [S7]. Roughly speaking, corresponding algorithms integrate over the range of possible (likely) population histories weighting by their compatibility with the observed data (likelihood). They proceed through a step by step chain of genealogies with slight random modification from one step to the next (topology, branch lengths, or parameter values). Such methods potentially use the full information in the data and primarily aim at parameter estimation, in particular the mutation rate and the effective size, which can in principle be disentangled with serially sampled data. More complex models include demographic changes, i.e., expansion dates in particular through the so-called skyline plot [S8]. However, such data driven simulation methods as well as rejection algorithms are not appropriate to assess the heterochrony driven bias, since they involve a distribution of trees biased toward the data compared to a strictly neutral, e.g. WF, distribution. Moreover, we suspect that there is often too little information in most datasets, especially for aDNA, to estimate the whole set of parameters reliably, indeed not all serially sampled data are "measurably evolving". For instance, on our application dataset, the correlation between the age of the sequences and the genetic distance from the most parsimonious ancestral state is hardly significant (see Figures S1, S2 above). Such methods are also computationally intensive, making it more difficult to run reliable analyses of their statistical properties which is thus virtually never completed on a reasonable number of simulation and range of conditions (e.g., there is little information about the robustness to details in the model assumptions [S9]). Finally they generally assume an absence of recombination or are rather inefficient when there is any (the likelihood surface is then very rugged). They require rather specialized users to get reliable meaningful results. Preliminary analyses on our example dataset suggest that it is hard to obtain repeatable realistic results, and in particular to estimate independently the mutation rate and the effective size (correlated estimates; results not shown).

A simple commonly used alternative to full MCMC likelihood methods in particular in the presence of recombination, is to survey summary statistics which relate to some key s aspects of the data at the expense of some loss of information (see the introduction of the paper for an overview of such statistics and the methods for a more specific list of the one illustrated here). Their distribution can be easily obtained empirically by simulation, including for heterochroneous data [S10]. The assessment of the statistical properties of these summary statistics is more feasible, has been largely investigated and they appear robust to the details of the model (see e.g. [S11]). The current trend is to combine several summary statistics in an Approximate Bayesian Computation framework (ABC, an extension of rejection methods [S12]) to estimate the parameters of the models.

Simulations:

For the simulation part, we conditioned the results on a given mutational parameter of the population $\theta=2N_e\mu$, assuming a haploid population with a given mutation rate (μ) and effective size (N_e of females for mtDNA data). However, for testing the outcome of each simulation or real dataset, we conditioned the tests on S, the observed number of polymorphic sites in the (simulated) dataset, assuming that the mutational parameter is generally unknown when applying the tests (as justified in [S11] and references therein). We used a nominal value for the tests of 5% on each side considering one-tailed tests (this should be generally the case in practice, at least for the F_{st} and Pearson's statistics).

A minimum of 10,000 simulations were run for each set of parameter values and 500 permutations were used in randomization tests (F_{st} and Pearson correlation test, greater numbers of permutations would have been too computationally intensive, given the large number of coalescent simulations on which the test is to be performed).

The algorithm proceeds as follow. Given a contemporaneous subset of size g (number of current tips of the tree) at time t, if the drawn coalescent time leads to a time older than the time t_i to the next subset, the event is cancelled and the process is started back from time t_i and an updated subset size of $g+n_i$. This procedure is appropriate because of the time Markovian property of coalescence: the probability to reach a common ancestor only depends on the current state and not on what happens before (going backward in time): given that no (further) common ancestry occurred at time t the probability to get one in the same generation is unchanged (only a function of the current sample and effective sizes). This algorithm is equivalent to that of Achaz and colleagues [S13] and the process described by Rodrigo [S4]. A similar adaptation of coalescent algorithm to serially sampled data is distributed in Serial Simcoal [S10], to be used in conjunction with Arlequin analysis software [S14] albeit the set of statistics provided is much more limited than the one we consider here (see Methods).

In practice, an estimate of the effective size is required to convert times in evolutionary scale. We used the previously available estimate of 660 KYA for the age of the MRCA [S15]. Under a Wright-Fisher, constant size model, this corresponds to an expected age of $(1–1/n)$ in units of $2N_e$ generations on a dataset of n=13 sequences, thus providing a straightforward moment estimate of N_e. It should have poor statistical properties. In practice, a more refined estimator of N_e should be used. It is noteworthy that this estimator paradoxically assumes an absence of heterochrony. In fact, a major limitation is that the estimate of N_e should take into account the heterochrony effect, which makes the argument somewhat circular (see equation (1;3)). To estimate the effect of heterochrony, we need a parameter estimate which should however be biased by heterochrony. It was argued that the time range was too small to show a detectable effect on the statistics (note however that the dataset of Hofreiter and colleagues [S15,S16] showed a smaller time range: 25–65 KYA). At any rate, this procedure can only underestimate the heterochrony effect and is therefore conservative. Moreover this problem can be solved when an independent estimate of the mutation rate is available (see equation (2;4)). Finally, we also used the likelihood method of Drummond and colleagues [S7] that takes into account the heterochrony to derive a MCMC Metropolis-Hasting estimate of the product of the effective size by the generation time (together with other parameters). When applied to our dataset on the subset of sequences with time estimates available using priors equivalent to those described in [S15], this provided an estimate of N_e compatible with the one used here (results not shown). Therefore, our estimate should not be grossly biased (in agreement with the results on Table 1). This appears adequate enough since our

intention here is to highlight the orders of magnitude in errors resulting from ignoring such a serial sampling effect and not to draw precise quantitative conclusions on the current dataset. Note however that it would be better, for statistical independency reasons, to use N_e estimates not relying on the dataset properties whenever possible (this issue is close to that of estimating θ from a dataset, assuming a neutral model and using this estimate to test the neutral model on the same data conditioning on this θ value; see [S17] and references therein for a discussion of related issues).

Uncertainty in the estimation of N_e was translated into uncertainty in time estimates. For the time uncertainty analyses, we allowed the age of N_e to vary uniformly within the limits of the posterior range estimated from [S15]. We applied an equivalent procedure for the generation time on their prior range (10–17 years). This approach tends to overestimate the range of uncertainty and should thus be conservative. A Bayesian-like type of approach taking into account the prior information on all parameters should be more appropriate in general. In the present application though, this may appear excessive given the weakness of the effects involved (Tables 2 and 3).

Supplementary References:

S1. Epperson BK (1999) Gene genealogies in geographically structured populations. Genetics 152:797-806.30.

S2. Barton NH, Depaulis F, Etheridge AE (2002) Neutral evolution in spatially continuous populations. Theor Popul Biol 61: 31-48.

S3. Forsberg R, Drummond AJ, Hein J (2005) Tree measures and the number of segregating sites in time-structured population samples. BMC Genet. 6:35.

S4. Rodrigo AG (1999) HIV evolutionary genetics. Proc Natl Acad Sci U S A 96:10559-10561.

S5. Liu X, Fu YX (2008) Summary statistics of neutral mutations in longitudinal DNA samples. Theor Popul Biol 74:56–67.

S6. Watterson GA (1975) On the number of segregation sites. Theor Popul Biol 7:256-276.

S7. Drummond AJ, Nicholls GK, Rodrigo AG, Solomon W (2002) Estimating mutation parameters, population history and genealogy simultaneously from temporally spaced sequence data. Genetics 161:1307-1320.

S8. Drummond AJ, Rambaut A, Shapiro B, Pybus OG (2005) Bayesian coalescent inference of past population dynamics from molecular sequences. Mol Biol Evol 22:1185-1192.

S9. Axelsson E, Willerslev E, Thomas M, Gilbert P, Nielsen R (2008) The effect of ancient DNA damage on inferences of demographic histories. Mol Biol Evol 25:2181-2187.

S10. Anderson CN, Ramakrishnan U, Chan YL, Hadly EA (2005) Serial SimCoal: a population genetics model for data from multiple populations and points in time. Bioinformatics 21:1733-1734.

S11. Depaulis F, Mousset S, Veuille M (2005) Detecting selective sweeps with haplotype tests. In: Nurminsky D, editor. Selective sweep. Georgetown, TX: Landes Bioscience. pp. 34-54.

S12. Beaumont MA, Zhang W, Balding DJ (2002) Approximate Bayesian computation in population genetics. Genetics 162:2025–2035.

S13. Achaz G, Palmer S, Kearney M, Maldarelli F, Mellors JW et al. (2004) A robust measure of HIV-1 population turnover within chronically infected individuals. Mol Biol Evol 21:1902–1912.

S14. Excoffier L, Laval G, Schneider S (2005) Arlequin ver. 3.0: An integrated software package for population genetics data analysis. Evol. Bioinform. Online 1:47-50.

S15. Hofreiter M, Capelli C, Krings M, Waits L, Conard N, et al. (2002) Ancient DNA analyses reveal high mitochondrial DNA sequence diversity and parallel morphological evolution of Late Pleistocene Cave Bears. Mol Biol Evol 19:1244-1250.

S16. Hofreiter M, Rabeder G, Jaenicke-Despres V, Withalm G, Nagel D, et al. (2004) Evidence for reproductive isolation between cave bear populations. Curr Biol 14:40-43.

S17. Depaulis F, Mousset S, Veuille M (2001) Haplotype tests using coalescent simulations conditional on the number of segregating sites. Mol Biol Evol 18:1136-1138.

S18. Wall JD (1999) Recombination and the power of statistical tests of neutrality. Genet Res 74:65-69.

S19. Bandelt HJ, Forster P, Röhl A (1999) Median-joining networks for inferring intraspecific phylogenies. Mol Biol Evol 16:37-48.
