# Supplementary figures and images for: Using Classical Population Genetics Tools with Heterochroneous Data: Time Matters!
Source: PLoS One. 2009 May 14;4(5):e5541. doi: 10.1371/journal.pone.0005541 (PMC2678253; doi:10.1371/journal.pone.0005541)

**A**

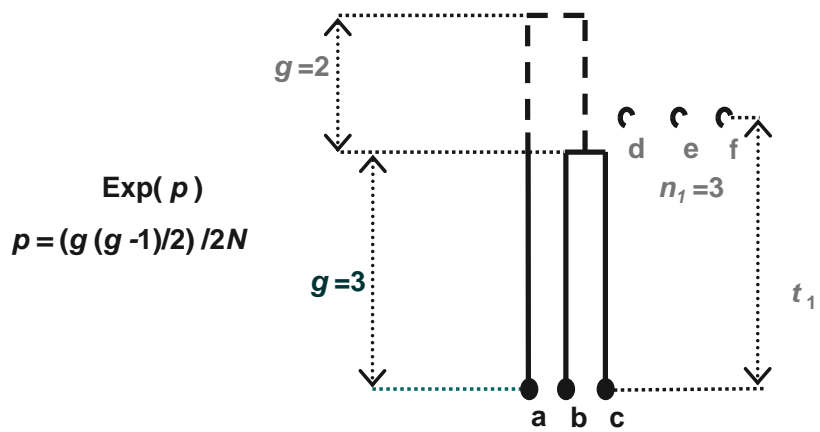

# B

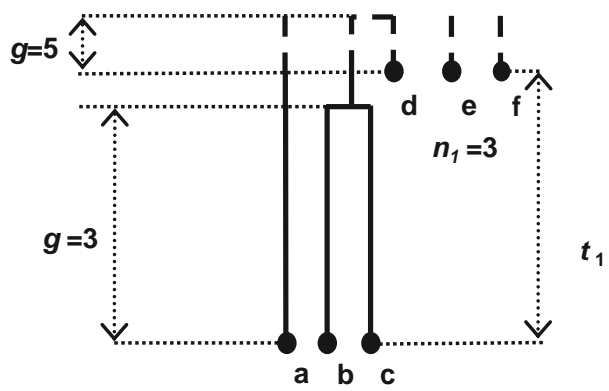

C

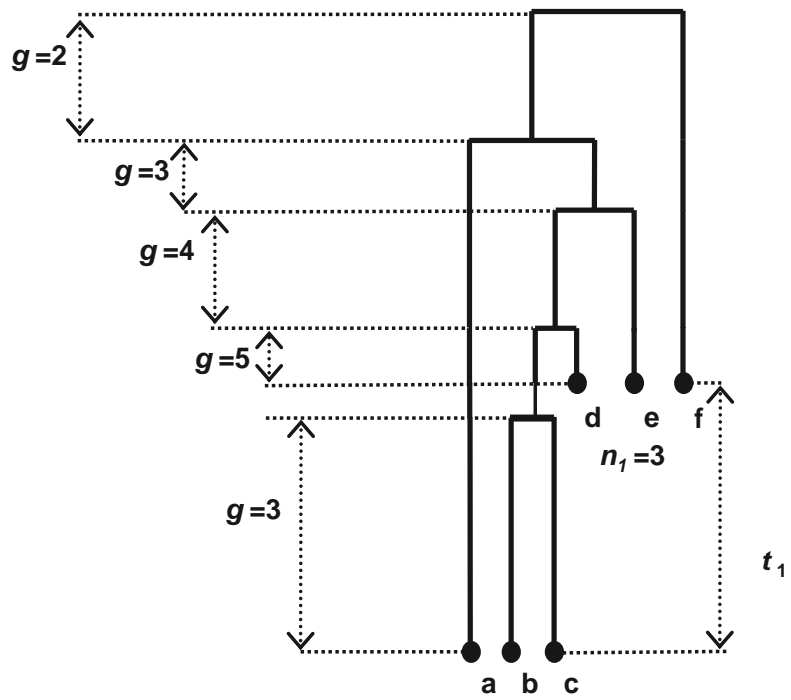

Supplement: Figure S1 — Algorithm for simulations of heterochroneous genealogies. The classical algorithm (exponential coalescent times) starts with the most recent subset (A); until a coalescent time exceeds the time to the next subset t 1; then (B) the event is cancelled and the algorithm starts back from time t 1 with a number of lineages g updated by adding n 1, until (C) the MRCA is reached. (0.08 MB PDF) [file pone.0005541.s002.pdf]

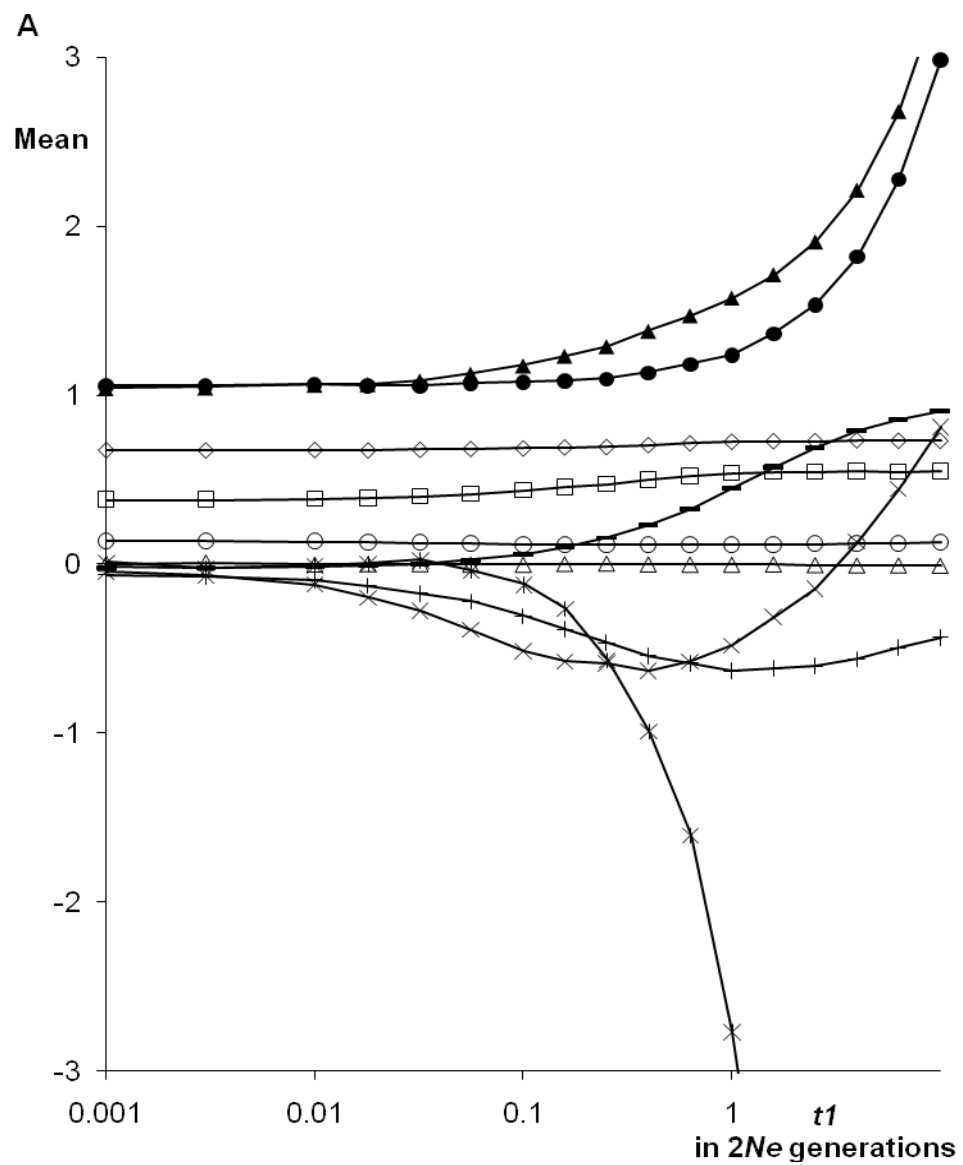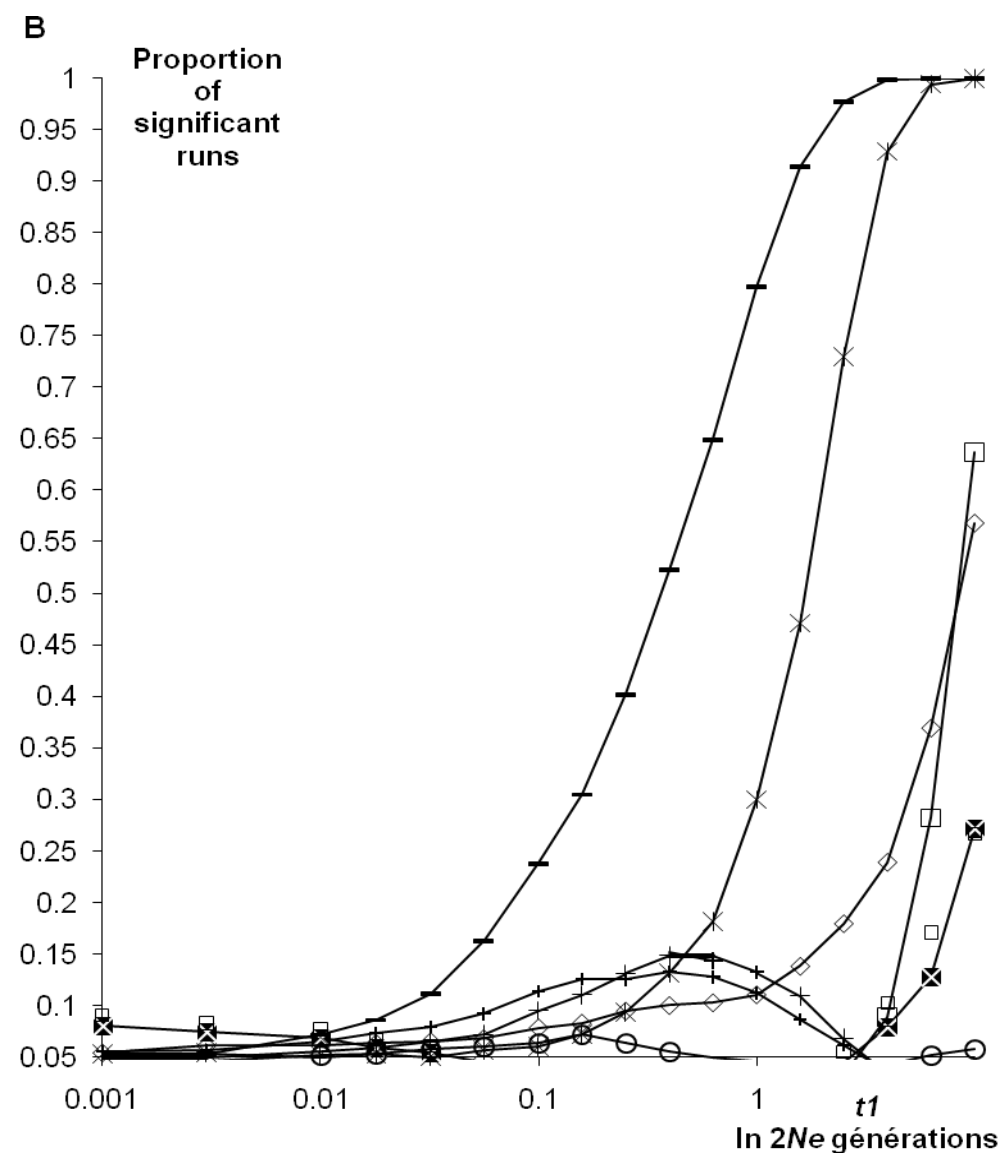

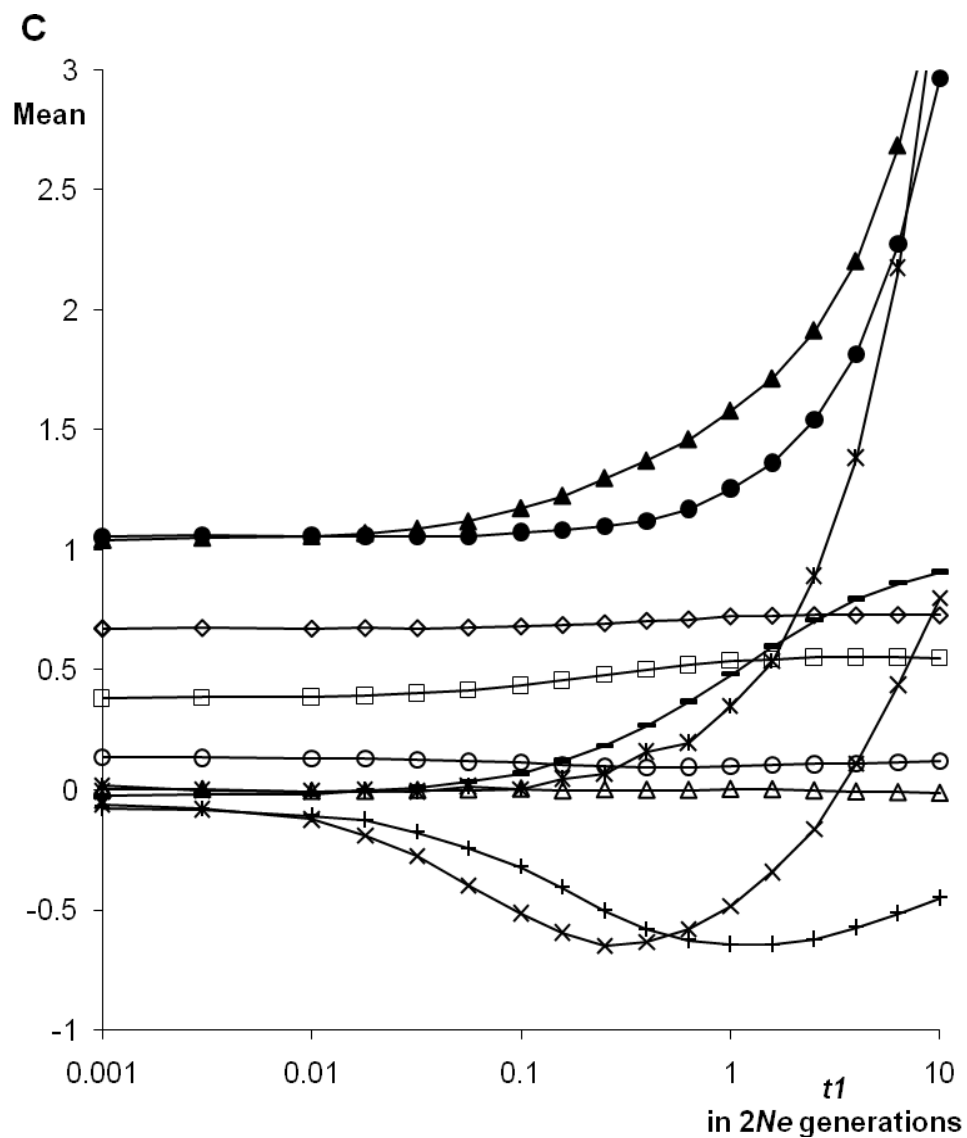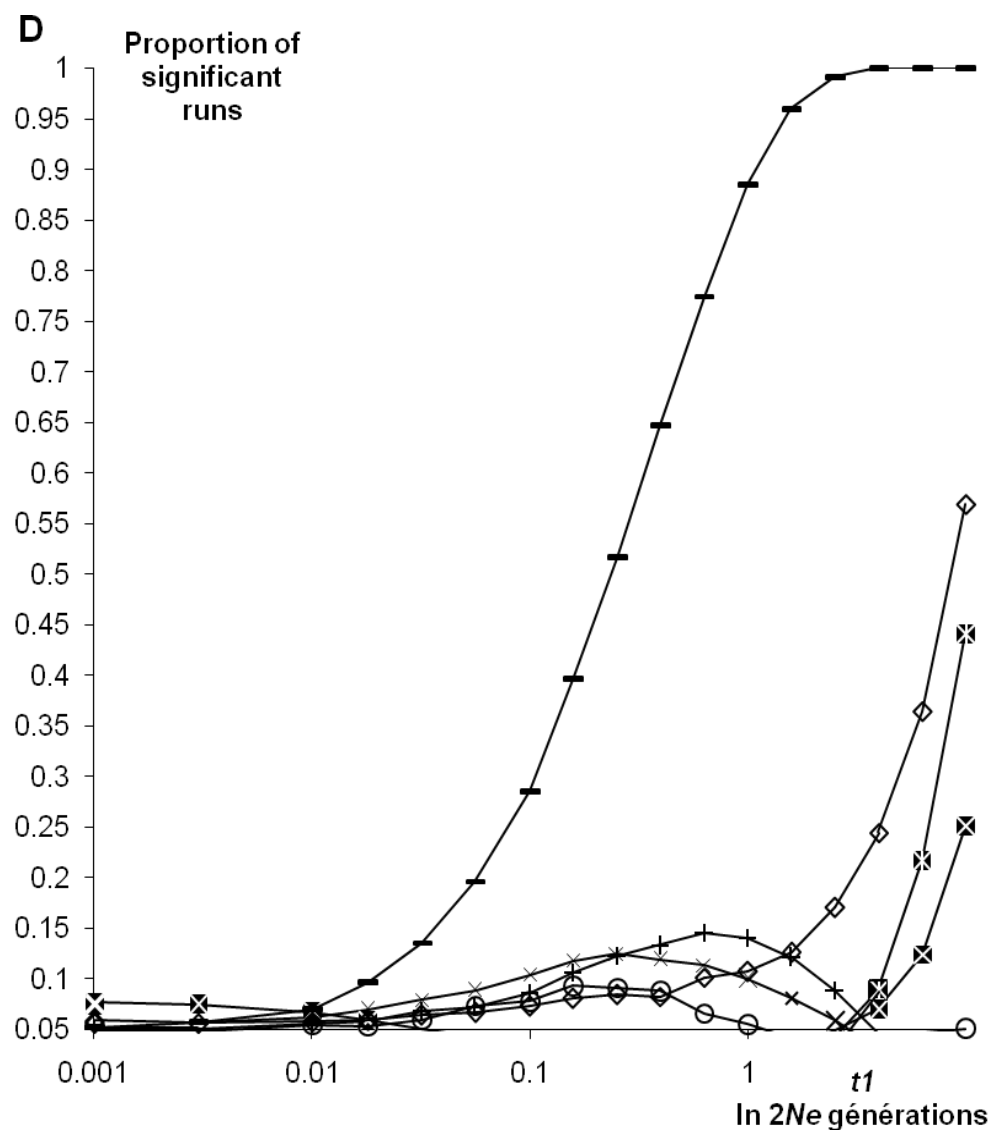

Supplement: Figure S2 — Effect of heterochrony on statistical tests as a function of time spacing. (A, B): 10% subset;(C, D) 90% subset (n 1 = 10 or 90, respectively, whole second population subsample in the Fst analysis). The X axis is expressed in units of 2Ne generations. Same labeling as in figure 2. The effects of other parameters such as total number of sequences in the dataset and polymorphism levels were investigated elsewhere [S18] and do not show noticeable interaction with the heterochrony effect (the effects described here simply appear stronger for larger datasets especially for increasing sample size since when the heterochrony range is limited and affects mostly the short external branches. (0.18 MB PDF) [file pone.0005541.s003.pdf]

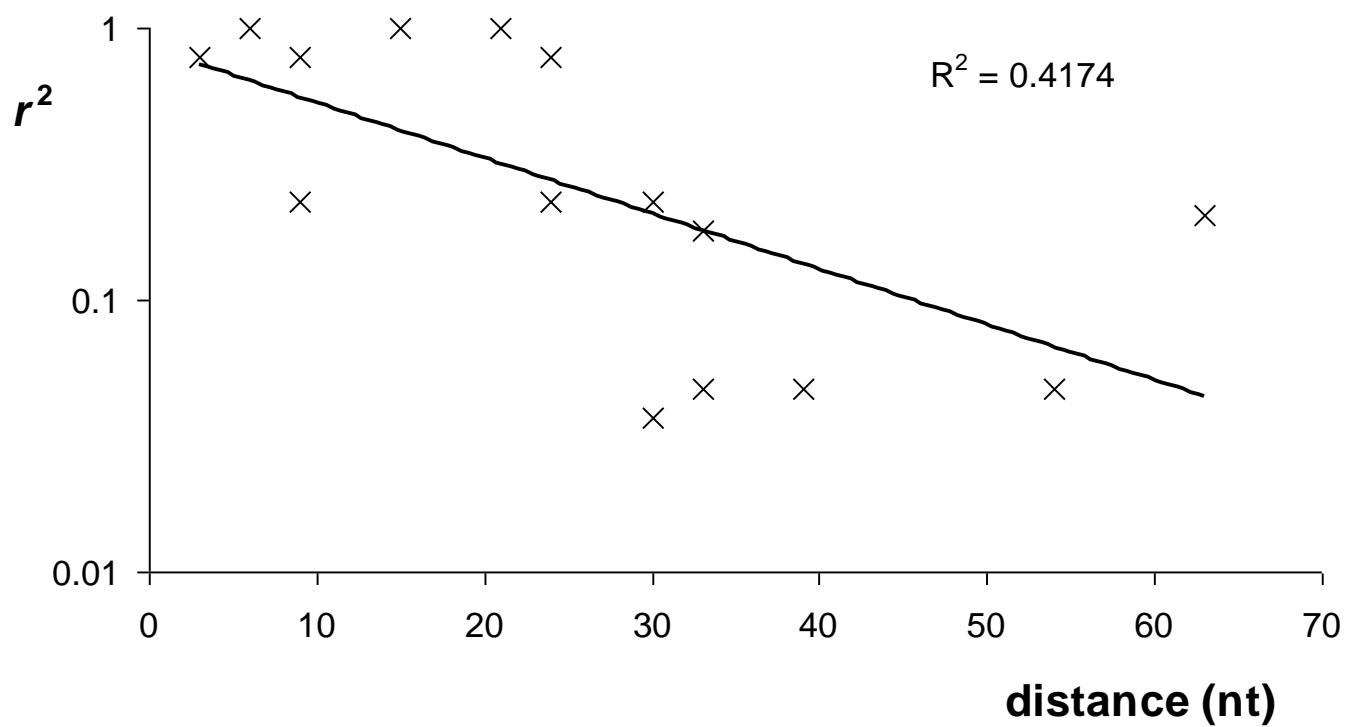

Supplement: Figure S5 — Allelic correlation (r 2) as a function of distance between informative sites in the Belgian Cave Bear subsample. The Y axis is on a log scale as an exponential relationship is approximately expected for the recombination effect (strictly, this corresponds to the expectation under a deterministic approximation). An exponential regression is shown for comparison. (0.06 MB PDF) [file pone.0005541.s006.pdf]

**A**

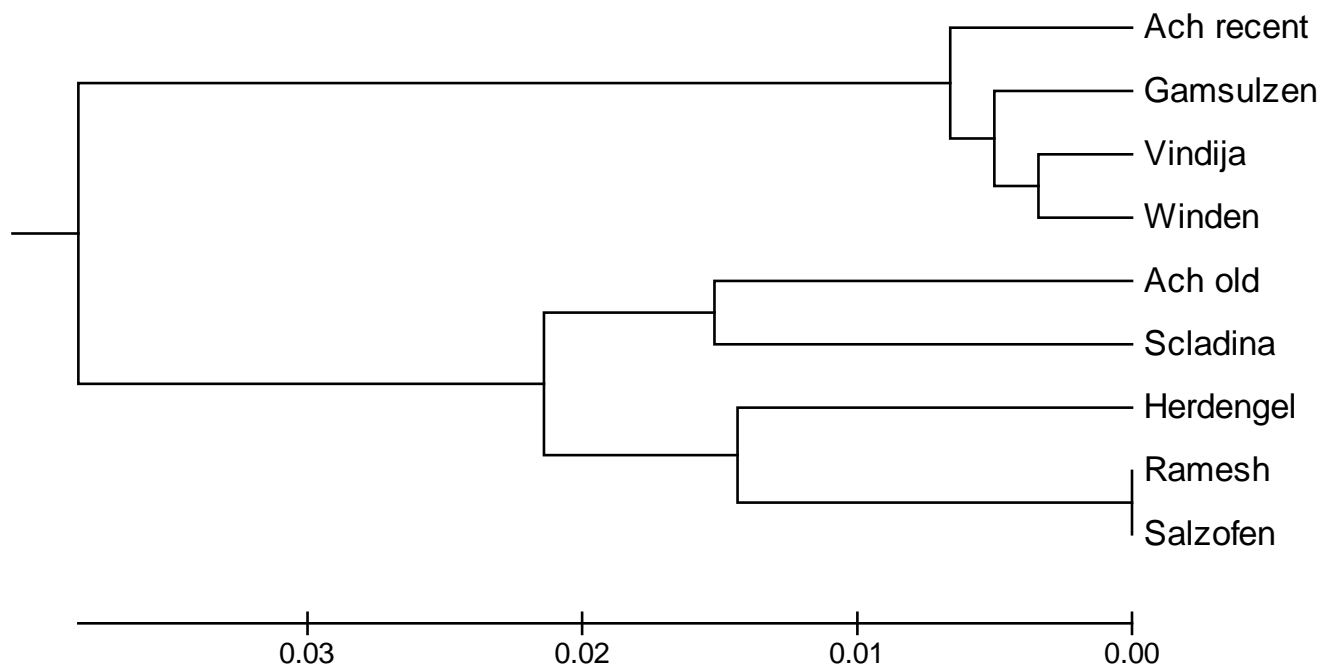

**B**

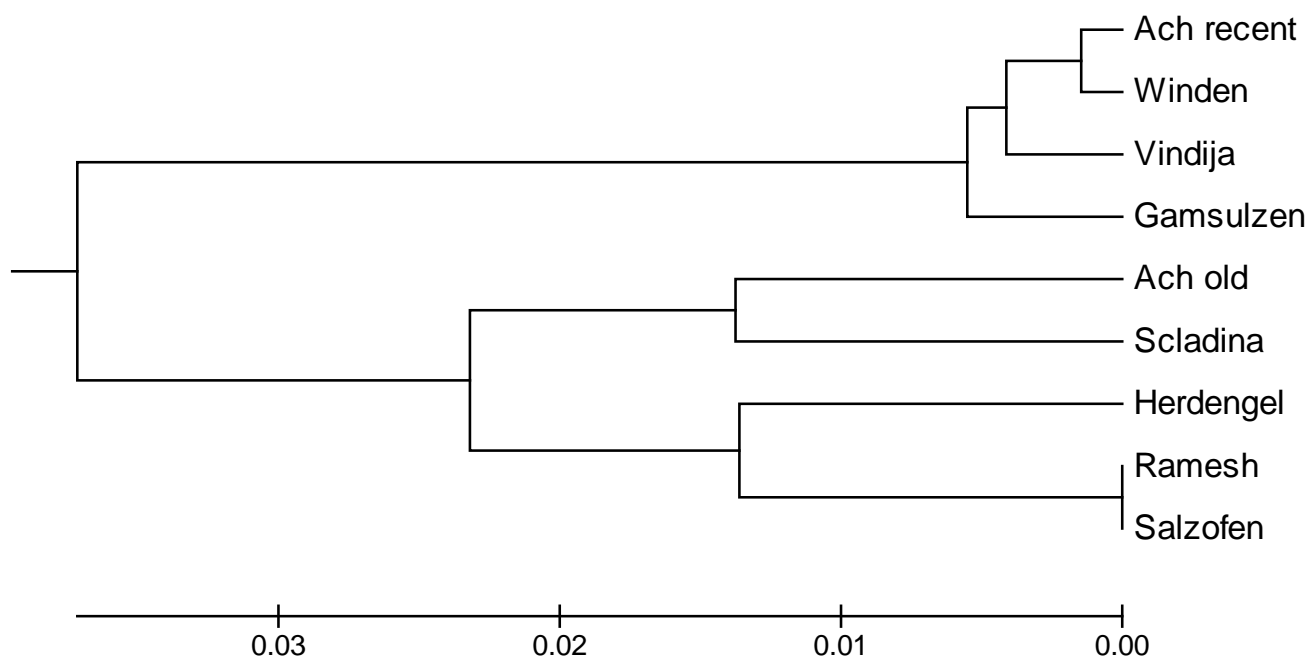

Supplement: Figure S6 — UPGMA tree between Cave Bear populations from pairwise distances. A: uncorrected. B: corrected for heterochrony. (0.06 MB PDF) [file pone.0005541.s007.pdf]
