# Supplementary material for: Using Classical Population Genetics Tools with Heterochroneous Data: Time Matters!
Source: PLoS One. 2009 May 14;4(5):e5541. doi: 10.1371/journal.pone.0005541 (PMC2678253; doi:10.1371/journal.pone.0005541)
Supplement: Figure S3 — Alignment of polymorphic sites of the Cave Bear dataset. For each site, the given number refers to the position relative to the first nucleotide of the sequence under Accession Number AY149238. Accession numbers are reported as sequence names. For haplotypes that stem from different non-overlapping sequences, a list of corresponding Accession numbers is given below the alignment. Haplotypes are referenced according to the following: AccessionNumberIfAvailable_Name_MinimumAge_MaximumAge_(Location. (0.13 MB PDF) [file pone.0005541.s004.pdf]

Total number of Sequences = 119

Total number of Sites = 322

```
Positions                                          111111111 1111222222 2222222222 2222222222 3
                                                  1224445555 5014666777 8899000011 2222223334 4445567789 1
                                                  2475670123 9329589012 2347345838 3456780271 3486753662 5
                                                  ********************************************************
Reference (most parsimonious ancestral state)     ???CCCTGTC CCCTCGCCCC AGTTGTCAAC TTTCGAACTG TGAAGTGCAA T
SalzofenOchsenhalt n=4                            ********************************************************
SO1_022_130_(SalzofenOchsenhalt)                  ???.T..AC. T????..... G...A..G.. ....A..T.A C..??????? ?
SO2_022_130_(SalzofenOchsenhalt)                  ???.T..AC. T????..... G...A..G.. ....A..T.A C..??????? ?
OH1_022_130_(SalzofenOchsenhalt)                  ???.T..AC. T????..... G...A..G.. ....A..T.A C..??????? ?
OH2_022_130_(SalzofenOchsenhalt)                  ???.T..AC. T????..... G...A..G.. ....A..T.A C..??????? ?
AchValley n=20                                    ********************************************************
AJ300176_HFels_030_031_(AchValley)                ???.T..ACT T......... G...A....T .C..A..TCA .......... .
AJ300170_Geis1_026_027_(AchValley)                ???.T.CACT T......... .....CT... ...T.G.TCA .......... .
AJ300171_Geis2_026_027_(AchValley)                ???.T.CACT T......... .....CT... ...T.G.TCA .......... .
geisenk3G_027_028_(AchValley)                     ???.T.CACT T????..... .....CT... ...T.G.TCA ...??????? ?
geisenk4G_027_028_(AchValley)                     ???.T.CACT T????..... .....CT... ...T.G.TCA ...??????? ?
geisenk5G_027_028_(AchValley)                     ???.T.CACT T????..... .....CT... ...T.G.TCA ...??????? ?
geisenk6S_025_026_(AchValley)                     ???.T.CACT T????..... .....CT... ...T.G.TCA ...??????? ?
geisenk7S_026_027_(AchValley)                     ???.T.CACT T????..... .....CT... ...T.G.TCA ...??????? ?
A251H_027_029_(AchValley)                         ???.T..AC. T????..... G...A....T .CC.A..T.A ...??????? ?
A252H_028_029_(AchValley)                         ???.T..AC. T????..... G...A....T .CC.A..T.A ...??????? ?
A253H_028_029_(AchValley)                         ???.T..AC. T????..... G...A....T .CC.A..T.A ...??????? ?
A254H_027_028_(AchValley)                         ???.T..AC. T????..... G...A....T .CC.A..T.A ...??????? ?
A255H_027_029_(AchValley)                         ???.T..AC. T????..... G...A....T .CC.A..T.A ...??????? ?
A256H_027_029_(AchValley)                         ???.T..AC. T????..... G...A....T .CC.A..T.A ...??????? ?
A257G_029_030_(AchValley)                         ???.T..AC. T????..... G...A....T .CC.A..T.A ...??????? ?
A271G_030_032_(AchValley)                         ???.T..ACT T????..... G...A....T .C..A..T.A ...??????? ?
A272G_037_039_(AchValley)                         ???.T..ACT T????..... G...A....T .C..A..T.A ...??????? ?
A273S_035_036_(AchValley)                         ???.T..ACT T????..... G...A....T .C..A..T.A ...??????? ?
A281G_033_034_(AchValley)                         ???.T..AC. T????..... G...A....T .C..A..T.A ...??????? ?
A282G_028_030_(AchValley)                         ???.T..AC. T????..... G...A....T .C..A..T.A ...??????? ?
Vindija n=12                                      ********************************************************
AJ300174_Vin1_033_034_(Vindija)                   ???.T.CACT T..C...... .....CT... ...T...TCA .......... .
AJ300175_Vin2_031_033_(Vindija)                   ???.T.CACT T......... .....CT... ...T...TCA ......A.G. .
vi11_041_044_(Vindija)                            ???.T.CACT T????..... .....CT... ...T...TCA ...??????? ?
vi12_047_051_(Vindija)                            ???.T.CACT T????..... .....CT... ...T...TCA ...??????? ?
vi13_023_024_(Vindija)                            ???.T.CACT T????..... .....CT... ...T...TCA ...??????? ?
vi14_022_023_(Vindija)                            ???.T.CACT T????..... .....CT... ...T...TCA ...??????? ?
vi15_022_051_(Vindija)                            ???.T.CACT T????..... .....CT... ...T...TCA ...??????? ?
Vi4_041_044_(Vindija)                             ???.T.CACT T????..... G....CT... ...T...TCA ...??????? ?
Vi31_044_047_(Vindija)                            ???.T.CACT T????..... G....CT... ......GTCA ...??????? ?
Vi32_042_045_(Vindija)                            ???.T.CACT T????..... G....CT... ......GTCA ...??????? ?
Vi21_038_040_(Vindija)                            ???.T.CACT T????..... G....CT... .......TCA ...??????? ?
Vi22_040_042_(Vindija)                            ???.T.CACT T????..... G....CT... .......TCA ...??????? ?
Scladina n=20                                     ********************************************************
AY149267_SC3500_030_040_(Scladina)                ???.T..AC. T....A.... G...A....T .C..A..T.A .......... .
AY149268_SC3800_030_040_(Scladina)                ???.T..AC. T....A.... G...A....T .C..A..T.A .......... .
AY149238_SC85F16_030_040_(Scladina)               TCC.T..AC. T...T.TTTT G......... .......T.A ......???? ?
SC92152_030_040_(Scladina)                        TCG.T..AC. T....A.... G.C.A....T .C..A..T.A ....A..... .
SC92386_030_040_(Scladina)                        ?????????? ?????????? ??..A....T .C..A..T.A .......T.. .
AY149243_SC92413_030_040_(Scladina)               TCGTTT.AC. T....A.... G.C.A....T .C..A..T.A ......???? ?
SC95456_030_040_(Scladina)                        TCG.TT.AC. TTT.?????? ??..A....T .C..A..T.A ......???? ?
AY149269_SC5300_040_045_(Scladina)                ?????????? ?????????? ??..A....T .C..A..T.A .......... .
AY149246_SC100801_080_120_(Scladina)              ?????????? ?????????? ??........ .......T.A ......???? ?
SC11600_080_120_(Scladina)                        CTGTT..AC. T...?????? ??........ .......T.A .........G .
AY149250_SC11700_080_120_(Scladina)               ?????????? ?????????? ??C.A....T .C..A..T.A ....A.???? ?
SC11800_080_120_(Scladina)                        ?????????? ?????????? ??.CA....T .C..A.GT.A .......... C
SC12400_080_120_(Scladina)                        TCG.T..AC. T...?????? ??..A....T .C..A..T.A ......???? ?
AY149255_SC12500_080_120_(Scladina)               ???TTT.AC. T...T..... G......... .......T.A ......???? ?
AY149256_SC13800_080_120_(Scladina)               ?????????? ?????????? ??........ .......T.A ......???? ?
AY149261_SCbrC5?02_090_130_(Scladina)             ?????????? ?....A.... .AC.A....T .C..A..T.A ......???? ?
AY149257_SC15400_090_130_(Scladina)               ?????????? ?????????? ??C.A....T .C..A..T.A ...G..???? ?
AY149258_SC15700_090_130_(Scladina)               ?????????? ?......... ......T.G. C..TA..TCA ..G...???? ?
AY149260_SC84G19_090_130_(Scladina)               ?????????? ?....A.... .AC.A....T .C..A..T.A ......???? ?
AY149259_SC84E16_090_130_(Scladina)               ?????????? ?....A.... .AC.A....T .C..A..T.A ......???? ?
Ramesch n=9                                       ********************************************************
AJ300166_Ram1_048_051_(Ramesch)                   ???.T..AC. T......... G...A..G.. ....A..T.A C......... .
AJ300167_Ram2_043_045_(Ramesch)                   ???.T..AC. T......... G...A..G.. ....A..T.A C......... .
Ram3_050_130_(Ramesch)                            ???.T..AC. T????..... G...A..G.. ....A..T.A C..??????? ?
Ram4_050_130_(Ramesch)                            ???.T..AC. T????..... G...A..G.. ....A..T.A C..??????? ?
Ram5_050_130_(Ramesch)                            ???.T..AC. T????..... G...A..G.. ....A..T.A C..??????? ?
Ram6_050_130_(Ramesch)                            ???.T..AC. T????..... G...A..G.. ....A..T.A C..??????? ?
Ram7_042_045_(Ramesch)                            ???.T..AC. T????..... G...A..G.. ....A..T.A C..??????? ?
Ram8_045_050_(Ramesch)                            ???.T..AC. T????..... G...A..G.. ....A..T.A C..??????? ?
Ram9_030_032_(Ramesch)                            ???.T..AC. T????..... G...A..G.. ....A..T.A C..??????? ?
Gamsulzen n=7                                     ********************************************************
Gam11_031_033_(Gamsulzen)                         ???.T.CACT T????..... .....CT... ...T...TCA ...??????? ?
Gam12_031_033_(Gamsulzen)                         ???.T.CACT T????..... .....CT... ...T...TCA ...??????? ?
Gam21_036_038_(Gamsulzen)                         ???.T.CACT T????..... G....CT... .......TCA ...??????? ?
```

Gam22_043_046_(Gamsulzen)	???.T.CACT T????..... G....CT... ....TCA ...??????? ?
Gam23_043_047_(Gamsulzen)	???.T.CACT T????..... G....CT... ....TCA ...??????? ?
Gam24_040_042_(Gamsulzen)	???.T.CACT T????..... G....CT... ....TCA ...??????? ?
Gam25_045_050_(Gamsulzen)	???.T.CACT T????..... G....CT... ....TCA ...??????? ?
**Winden *n*=7**	***
wind1_022_130_(Winden)	???.T.CACT T????..... .....CT... ...T...TCA ...??????? ?
wind2_022_130_(Winden)	???.T.CACT T????..... .....CT... ...T...TCA ...??????? ?
wind3_022_130_(Winden)	???.T.CACT T????..... .....CT... ...T...TCA ...??????? ?
wind4_022_130_(Winden)	???.T.CACT T????..... .....CT... ...T...TCA ...??????? ?
wind5_022_130_(Winden)	???.T.CACT T????..... .....CT... ...T...TCA ...??????? ?
wind6_022_130_(Winden)	???.T.CACT T????..... .....CT... ...T...TCA ...??????? ?
wind7_022_130_(Winden)	???.T.CACT T????..... .....CT... ...T...TCA ...??????? ?
**Herdengel *n*=8**	***
HD1_055_065_(Herdengel)	???.T.CACT T????..... G....CT... ...T...TCA ...??????? ?
HD2_055_065_(Herdengel)	???.T.CACT T????..... G....CT... ...T...TCA ...??????? ?
HD3_060_130_(Herdengel)	???.T..AC. T????..... G...A..G.. ....T.A C..??????? ?
HD4_060_130_(Herdengel)	???.T..AC. T????..... G...A..G.. ....T.A C..??????? ?
HD5_060_130_(Herdengel)	???.T..AC. T????..... G...A..... ....A..T.A ...??????? ?
HD6_060_130_(Herdengel)	???.T..AC. T????..... G...A..... ....A..T.A ...??????? ?
HD7_060_130_(Herdengel)	???.T..AC. T????..... G...A..G.. ...T...T.A ...??????? ?
HD8_060_130_(Herdengel)	???.T..AC. T????..... G...A..... ....A..T.A ...??????? ?
**Schwabenreith *n*=3**	***
SW1_072_130_(Schwabenreith)	???.T..AC. T????..... G...A..... ....T.A ...??????? ?
SW2_072_130_(Schwabenreith)	???.T..AC. T????..... G...A..G.. ....A..T.A ...??????? ?
SW3_072_130_(Schwabenreith)	???.T..AC. T????..... G...A..G.. ....A..T.A ...??????? ?
**Schreiberwand *n*=2**	***
SCR1_022_130_(Schreiberwand)	???.T..AC. T????..... G...A..G.T ....A..T.A C..??????? ?
SCR2_022_130_(Schreiberwand)	???.T..AC. T????..... G...A..G.T ....A..T.A C..??????? ?
**Cova Linares *n*=2**	***
AY149272_CLB_030_040_(CovaLinares)	???.T..AC. T....A.... G...A....T .CC.A..T.A ....
AY149271_CLA_030_040_(CovaLinares)	???.T..AC. T....A.... G...A....T .CC.A..T.A ....
**Brettstein *n*=2**	***
Bst1_022_130_(Brettstein)	???.T..AC. T????..... G......... ....T.A C..??????? ?
Bst2_022_130_(Brettstein)	???.T..AC. T????..... G...A..G.. ....A..T.A C..??????? ?
**Mixnitz *n*=2**	***
mixn1_022_130_(Mixnitz)	???.T.CACT T????..... .....CT... ...T...TCA ...??????? ?
mixn2_022_130_(Mixnitz)	???.T.CACT T????..... .....CT... ...T...TCA ...??????? ?
**Liegloch *n*=2**	***
lieg1_022_130_(Liegloch)	???.T.CACT T????..... .....CT... ...T...TCA ...??????? ?
lieg2_022_130_(Liegloch)	???.T.CACT T????..... .....CT... ...T...TCA ...??????? ?
**Hartelsgraben *n*=2**	***
hart1_022_130_(Hartelsgraben)	???.T.CACT T????..... .....CT... ...T...TCA ...??????? ?
hart2_022_130_(Hartelsgraben)	???.T.CACT T????..... .....CT... ...T...TCA ...??????? ?
**Slovenia *n*=2**	***
kris1_022_130_(Slovenia)	???.T.CACT T????..... .....CT... ...T...TCA ...??????? ?
kris2_022_130_(Slovenia)	???.T.CACT T????..... .....CT... ...T...TCA ...??????? ?
**One haplotype per location**	***
WK_022_130_(Wildkirchli)	???.T..AC. T????A.... G...A....T .C..A..T.A ...??????? ?
AY149264_Gigny189F3_022_130_(Gigny)	TCG.T..AC. T....A.... G...A....T .C..A..T.A ......???? ?
AJ300173_022_130_(PotockaZijalka)	???.T.CACT T......... .....CT... ...T...TCA ....
AJ300168_Gailenr_034_035_(Zoolithen)	???.T..AC. T....A.... ....A..... .C..A..T.A ....
AJ300169_Contur_043_046_(Conturines)	???.T..AC. T......... G........T ....T.A ....
AJ300172_Nix_026_028_(Nixloch)	???.T.CACT T......... .....CT... ...T...TCA ....
BB3_022_130_(Brieglersberg)	???.T..AC. T????..... G......... ....T.A C..??????? ?
APH_022_130_(Sulzfluh)	???.T..AC. T????..... G.....T... ...T...T.A ...??????? ?
AJ300177_GMer_035_036_(GrotteMerveilleuse)	???.T..AC. T......... G......... ...T...T.A ....
AY149266_TAB2_035_045_(Prelétang)	TCG.T..AC. T......... G......... ....T.A ......???? ?
AY149273_TAB15_025_035_(BalmeACollomb)	???.T..AC. T....A.... G...A..... .C..A..T.A ....
AZE_080_130_(Aze)	?????????? ?????????? ??........ ....T.A .....C.... .
AY149270_47910_025_035_(Mialet)	?????????? ?????????? ??..A....T .C..A..T.A .A........ .
AY149265_JAL104_022_130_(Mokrav)	?????????? ?????????? ??C.A....T .C..A..T.A ......???? ?
