# Supplementary material for: Using Classical Population Genetics Tools with Heterochroneous Data: Time Matters!
Source: PLoS One. 2009 May 14;4(5):e5541. doi: 10.1371/journal.pone.0005541 (PMC2678253; doi:10.1371/journal.pone.0005541)
Supplement: Figure S4 — Median-joining Haplotype network [S19] of the Cave Bear dataset. The sizes of the nodes are proportional to their frequencies. Each location is indicated by different colors. The most parsimonious ancestral state (“reference” in figure S1) is boxed in green. The average (or minimum whenever the average could not be computed) time to the sequenced is boxed in grey near the nodes. Correlation between the average age and the minimum number of mutational steps from the ancestral state: r 2 = 0.04*. The correlation between the age of the sequences and the genetic distance from the most parsimonious ancestral state is hardly significant, suggesting that there was too little information to estimate a whole set of parameters reliably under a full MCMC likelihood framework (see text S1). Indeed when we tried to apply the likelihood method of Drummond and colleagues [S7] to the data it was not able to disentangle the effective size from the mutation rate (highly correlated posterior distribution and we needed to provide an independent estimate of the mutation rate to estimate the effective size properly. We therefore did not rely on such approaches to assess the heterochrony driven bias. (0.12 MB PDF) [file pone.0005541.s005.pdf]

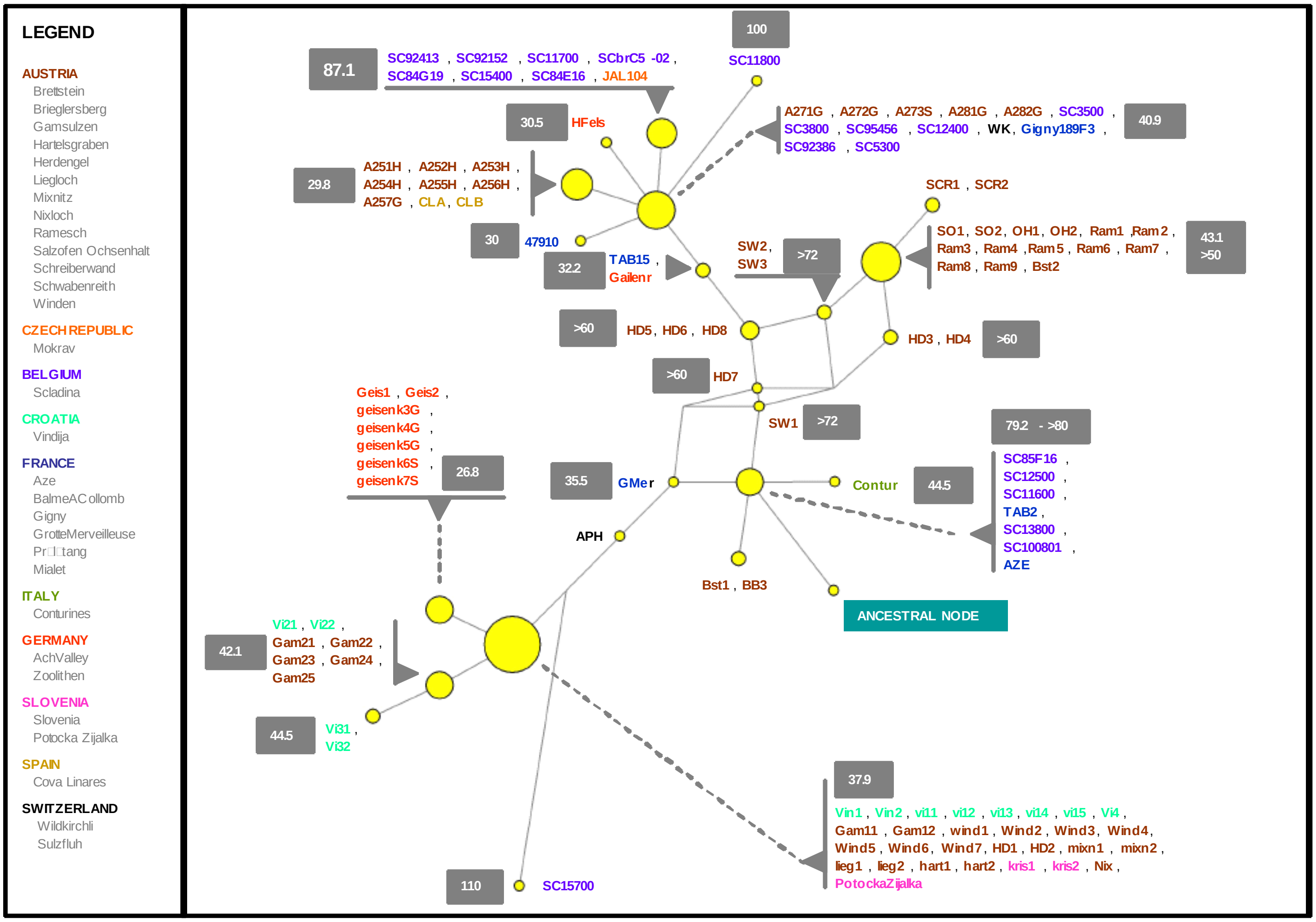
LEGEND
AUSTRIA
Brettstein
Brieglersberg
Gamsulzen
Hartelsgraben
Herdengel
Liegloch
Mixnitz
Nixloch
Ramesch
Salzofen Ochsenhalt
Schreiberwand
Schwabenreith
Winden
CZECH REPUBLIC
Mokrav
BELGIUM
Scladina
CROATIA
Vindija
FRANCE
Aze
BalmeACollomb
Gigny
GrotteMerveilleuse
Pr□l□tang
Mialet
ITALY
Conturines
GERMANY
AchValley
Zoolithen
SLOVENIA
Slovenia
Potocka Zijalka
SPAIN
Cova Linares
SWITZERLAND
Wildkirchli
Sulzfluh
87.1
SC92413 , SC92152 , SC11700 , SCbrC5 -02 , SC84G19 , SC15400 , SC84E16 , JAL104
100
SC11800
30.5
HFels
A271G , A272G , A273S , A281G , A282G , SC3500 , SC3800 , SC95456 , SC12400 , WK, Gigny189F3 , SC92386 , SC5300
40.9
29.8
A251H , A252H , A253H , A254H , A255H , A256H , A257G , CLA, CLB
SCR1 , SCR2
30
47910
32.2
TAB15 , Gailenr
SW2, SW3
>72
SO1, SO2, OH1, OH2, Ram1 ,Ram2 , Ram3 , Ram4 ,Ram5 , Ram6 , Ram7 , Ram8 , Ram9 , Bst2
43.1
>50
>60
HD5, HD6 , HD8
HD3 , HD4
>60
>60
HD7
Geis1 , Geis2 , geisenk3G , geisenk4G , geisenk5G , geisenk6S , geisenk7S
26.8
SW1
>72
79.2 - >80
35.5
GMer
Contur
44.5
SC85F16 , SC12500 , SC11600 , TAB2 , SC13800 , SC100801 , AZE
APH
Bst1 , BB3
ANCESTRAL NODE
42.1
Vi21 , Vi22 , Gam21 , Gam22 , Gam23 , Gam24 , Gam25
44.5
Vi31 , Vi32
37.9
Vin1 , Vin2 , vi11 , vi12 , vi13 , vi14 , vi15 , Vi4 , Gam11 , Gam12 , wind1 , Wind2 , Wind3, Wind4, Wind5 , Wind6, Wind7, HD1 , HD2 , mixn1 , mixn2 , lieg1 , lieg2 , hart1 , hart2 , kris1 , kris2 , Nix , PotockaZijalka
110
SC15700
