# Supplementary material for: Using Classical Population Genetics Tools with Heterochroneous Data: Time Matters!
Source: PLoS One. 2009 May 14;4(5):e5541. doi: 10.1371/journal.pone.0005541 (PMC2678253; doi:10.1371/journal.pone.0005541)
Supplement: Table S1 — List of Accession Numbers for sequences that stem from different non-overlapping PCR fragments. Some samples could be associated with an identical Accession number as they have been reported to exhibit identical haplotypes. (0.14 MB PDF) [file pone.0005541.s008.pdf]

SO1	AJ809306	AJ809228		
SO2	AJ809307	AJ809229		
OH1	AJ809308	AJ809230		
OH2	AJ809309	AJ809231		
geisenk3G	AJ809240	AJ809162		
geisenk4G	AJ809241	AJ809163		
geisenk5G	AJ809242	AJ809164		
geisenk6S	AJ809242	AJ809164		
geisenk7S	AJ809242	AJ809164		
A251H	AJ809245	AJ809169		
A252H	AJ809245	AJ809169		
A253H	AJ809246	AJ809168		
A254H	AJ809247	AJ809169		
A255H	AJ809248	AJ809170		
A256H	AJ809249	AJ809171		
A257G	AJ809250	AJ809172		
A271G	AJ809251	AJ809173		
A272G	AJ809252	AJ809174		
A273S	AJ809253	AJ809175		
A281G	AJ809255	AJ809177		
A282G	AJ809256	AJ809178		
vi11	AJ809274	AJ809196		
vi12	AJ809275	AJ809197		
vi13	AJ809276	AJ809198		
vi14	AJ809277	AJ809199		
vi15	AJ809278	AJ809200		
vi4	AJ809279	AJ809201		
vi31	AJ809280	AJ809202		
vi32	AJ809281	AJ809203		
vi21	AJ809282	AJ809204		
vi22	AJ809283	AJ809205		
SC92152	AY149240	AY149239	AH012172	
SC92386	AY149242	AY149241	AH012173	
SC95456	AY149245	AY149244	AH012174	
SC11600	AY149249	AY149248	AY149247	AH012175
SC11800	AY149252	AY149251	AH012176	
SC12400	AY149254	AY149253	AH012177	
Ram3	AJ809311	AJ809233		
Ram4	AJ809312	AJ809234		
Ram5	AJ809313	AJ809235		
Ram6	AJ809314	AJ809236		
Ram7	AJ809315	AJ809237		
Ram8	AJ809316	AJ809238		

Ram9	AJ809317	AJ809239	
Gam11	AJ809284	AJ809206	
Gam12	AJ809285	AJ809207	
Gam21	AJ809286	AJ809208	
Gam22	AJ809287	AJ809209	
Gam23	AJ809288	AJ809210	
Gam24	AJ809289	AJ809211	
Gam25	AJ809290	AJ809212	
wind1	AJ809291	AJ809213	
wind2	AJ809292	AJ809214	
wind3	AJ809293	AJ809215	
wind4	AJ809294	AJ809216	
wind5	AJ809295	AJ809217	
wind6	AJ809296	AJ809218	
wind7	AJ809297	AJ809219	
HD1	AJ809257	AJ809179	
HD2	AJ809258	AJ809180	
HD3	AJ809259	AJ809181	
HD4	AJ809260	AJ809182	
HD5	AJ809261	AJ809183	
HD6	AJ809262	AJ809184	
HD7	AJ809263	AJ809185	
HD8	AJ809264	AJ809186	
SW1	AJ809267	AJ809189	
SW2	AJ809268	AJ809190	
SW3	AJ809269	AJ809191	
SCR1	AJ809265	AJ809187	
SCR2	AJ809266	AJ809188	
Bst1	AJ809271	AJ809193	
Bst2	AJ809272	AJ809194	
mixn1	AJ809298	AJ809220	
mixn2	AJ809299	AJ809221	
lieg1	AJ809300	AJ809222	
lieg2	AJ809301	AJ809223	
hart1	AJ809302	AJ809224	
hart2	AJ809303	AJ809225	
kris1	AJ809304	AJ809227	
kris2	AJ809305	AJ809226	
WK	AJ809232	AJ809310	
BB3	AJ809270	AJ809192	
APH	AJ809273	AJ809195	
AZE	AY149263	AY149262	AH012178
